# An eco-epidemiological model for malaria with *Microsporidia MB* as bio-control agent

**DOI:** 10.1007/s40808-025-02322-1

**Published:** 2025-04-16

**Authors:** Charlène N. T. Mfangnia, Henri E. Z. Tonnang, Berge Tsanou, Jeremy Keith Herren

**Affiliations:** 1 International Centre of Insect Physiology and Ecology (icipe), P.O. Box : 30772, Nairobi 00100, Kenya; 2 Department of Mathematics and Computer Science, Faculty of Science, University of Dschang, P.O. Box : 67, Dschang, Cameroon; 3 School of Agricultural, Earth, and Environmental Sciences, University of KwaZulu-Natal, Pietermaritzburg 3209, South Africa; 4 International Institute of Tropical Agriculture (IITA), PMB, 5320 Oyo Road, Idi-Oshe Ibadan, Nigeria; 5 Department of Mathematics and Applied Mathematics, University of Pretoria, Pretoria 0002, South Africa

**Keywords:** *Microsporidia MB*, Malaria symbiotic control, Vertical and horizontal transmission, Mathematical modelling

## Abstract

*Microsporidia MB* is an endosymbiont which naturally infects *Anopheles* mosquitoes. Due to its ability to block *Plasmodium* transmission, it shows potential as a bio-based agent for the control of malaria. Its self-sustainability is promising, as it can spread through both vertical and horizontal transmissions. However, its low prevalence in mosquito populations remains a challenge. We develop an eco-epidemiological mathematical model describing the co-dynamics of *Microsporidia MB* (within mosquito population) and malaria (within human population). The model is used to assess the potential of *Microsporidia MB*-infected mosquitoes on the control of malaria infection. The results on the basic reproduction numbers, the stability of the equilibria, and the existence of bifurcations are obtained, providing conditions for the extinction and persistence of *MB*-infected mosquitoes. We highlight relevant threshold parameters for the elimination and persistence of *MB*-infected mosquitoes and malaria-infected individuals. Using real data from Kenya, we found that, given a horizontal transmission rate between 0 and 0.5, a minimum vertical rate of 0.55 is required to avoid extinction of *MB*-infected mosquitoes. The predicted prevalence of *MB*-infected mosquitoes using transmission rates reported from lab experiments align with the observed low prevalence of *MB*-infected mosquitoes in the field, thereby validating our model and results. Finally, predictions indicate that increasing *MB* mosquito infection could effectively control malaria, with target prevalence varying by region: 15% in Highland, 40% on the coast, and 70% in the Lake region. This study offers insights into the use of bio-based vector population replacement solutions to reduce malaria incidence in regions where *Microsporidia MB* is prevalent.

## Introduction

Malaria remains a pressing health challenge, requiring the adoption of a dual strategy encompassing both effective drug use and the control of mosquito vector populations. This comprehensive approach is pivotal in the ongoing fight against malaria. Insecticide-based strategies, particularly long-lasting insecticide-treated nets (LLINs) and indoor residual spraying (IRS) have played a significant role in curtailing malaria transmission between 2005 and 2015 (WHO 2022). These strategies rely predominantly on the use of a class of insecticides named *pyrethroid* (Oladipo et al 2022; Mohammed-Awel et al 2020). However, the efficacy of these insecticide-based strategies is increasingly compromised by the emergence of *pyrethroid* resistance (Oladipo et al 2022; Mohammed-Awel et al 2020). This resistance has contributed to a worrying increase in malaria cases and fatalities since 2019 (WHO 2022), highlighting a pressing need for alternative strategies.

Vector population replacement emerges as a novel approach to address the waning effectiveness of traditional insecticide-based methods. This innovative technique holds promise in the ongoing effort to effectively manage and control malaria, and the use of symbiont-infected mosquitoes offers a promising approach. The key to this approach is the use of self-sustaining symbionts that can be transmitted within mosquito populations. Effective symbiont-based techniques include genetically modified organisms like *Pantoea agglomerans* (Wang and Zou 2019; Kotnis and Kuri 2016), and *Serratia AS1* (Wang and Zou 2019), as well as natural variants such as *Asaia* (Favia et al 2008) and *Microsporidia MB*, the latter being naturally present in Anopheles mosquitoes (Herren et al 2020; Bukhari et al 2022; Nattoh et al 2021). The focus is shifting towards natural symbionts like *Microsporidia MB* for his inherent advantage and minimal ecological concerns as a stable and sustainable agent against malaria.

Found in *Anopheles arabiensis* and *Anopheles funestus* in Kenya (prevalence of 0–15%), and in *Anopheles coluzzi* (Akorli et al 2021) in Ghana, this microsporidian species belongs to the group of fungi. It shows superior impairment levels and vertical transmission efficiency compared to other previously discovered microsporidian impairing *Plasmodium* transmission such as *Nosema stegomyiae* and *Vavraia culcis* (Herren et al 2020). Offsprings of *MB*-infected females exhibit a symbiont presence rate of 45–100%. Post-exposure to *Plasmodium* *falciparum*, a reduction in *Plasmodium* oocysts and impaired colonization of sporozoites in the salivary glands were observed. Sexual transmission of *MB* was confirmed in mixed-infection experiments, with horizontal transmission rates of 56% from *MB*-infected males to uninfected females and 33% from infected females to uninfected males (Herren et al 2020). Despite a slightly faster development from egg to adult, mosquito fitness and longevity remained unaffected. Thanks to these promising properties, *MB* emerges as a potentially cost-effective, safe, and eco-friendly malaria control tool. To understand its impact on wild mosquito populations and malaria dynamics, and to estimate the achievable malaria control levels based on its prevalence, mathematical modelling holds a prominent place.

In the area of bio-agent use for controlling mosquito-borne diseases, various mathematical models have been developed to understand the interactions between wild and symbiont-infected mosquitoes across different strains. For instance, Ogunlade et al (2020) analyzed the dynamics between *wAu-Wolbachia*-infected and uninfected Aedes Aegypti mosquitoes. Similarly, Campo-Duarte et al (2018) explored interactions involving wild and *wMelPop-Wolbachia*-infected mosquitoes, and Li and Wan (2019), aimed to determine conditions for successful *Wolbachia* establishment in Aedes Aegypti populations. In the same vein, Florez et al (2023) assessed the conditions for establishment in the population of *Anopheles* *Albimanus*. Additionally, the impact of *Wolbachia* on dengue dynamics has been a significant area of research. In this regard, Hughes and Britton (2013) found that *Wolbachia* could control dengue if the basic reproduction number is manageable or, at minimum, reduce transmission. Ndii et al (2015) incorporated seasonal factors in their study, also affirming *Wolbachia*’s beneficial effects on dengue transmission. Moreover, Schraiber et al (2012) provided a model for spatial spread and establishment of *Wolbachia*-infected mosquitoes using a continuous time reaction-diffusion approach. Furthermore, Wang and Zou (2019) developed a climate-based malaria model that included both vertical and horizontal transmission, offering a threshold for the establishment of engineered *Serratia* bacteria in the mosquito population.

The present study builds on foundational research that has explored various aspects of *Microsporidia* dynamics, including gender demographics, horizontal and vertical transmission, and sex-structured populations (Heffernan et al 2014; Podder and Gumel 2009; Campo-Duarte et al 2018). Notably, previous studies have addressed factors such as differences in horizontal transmission probabilities between males and females, the once-only mating behaviour of females, and female sexual preferences (Heffernan et al 2014; Qu et al 2018; Patinvoh and Susu 2014). Given the recent identification of *Microsporidia MB* and its potential in malaria control, there is a significant gap in mathematical models that analyze its spread within mosquito populations. This study aims to fill this gap, offering insights into the potential impact of *Microsporidia MB* on the dynamics of malaria transmission, and is organized as follows.

The first section introduces the eco-epidemiological model that couples four populations (mosquito, *Microsporidia MB*, *Plasmodium* and humans) in the presence of malaria infection. The second section presents the key results of the mathematical analysis, which include the threshold numbers that determine malaria extinction or persistence, both in the absence and in the presence of the symbiont. The third section discusses the implications of the results, especially focusing on how the introduction of *Microsporidia MB* could alter the epidemiology and control strategies of malaria. The fourth section explores the impact of the incubation period on malaria dynamics, a factor omitted in the primary model formulation. Finally, the fifth section summarizes the results and concludes the study with a discussion of our findings, highlighting contributions to malaria research, acknowledging limitations, and suggesting avenues for future investigation.

## Method and models

**Fig. 1:**
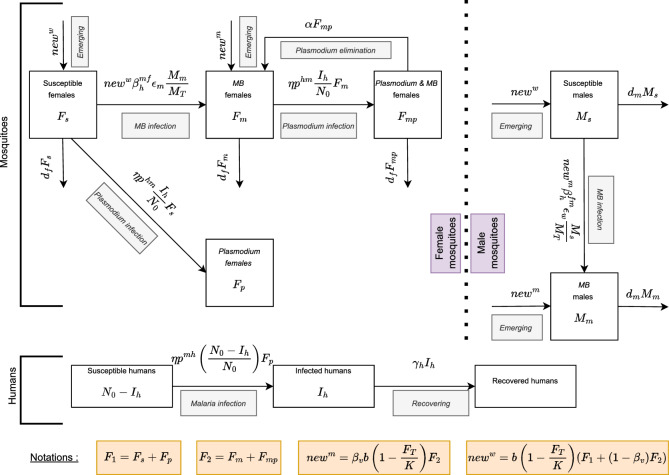
Flow diagram summarizing the interactions between humans, *Anopheles mosquitoes*, *Plasmodium* and *Microsporidia MB* as described in model (1)

The developed model couples an ecological model that describes the dynamics of *Microsporidia MB* in the mosquito population and an epidemiological model that describes the dynamics of malaria in the absence of *Microsporidia MB*. More importantly, the model accounts for the *Plasmodium* transmission-blocking ability associated with *MB* infection. The method involves the formulation and mathematical analysis of a system of differential equations which is developed based on the mosquito lifecycle, the spreading pathways of *Microsporidia MB* in mosquitoes, the malaria infection in humans, and a set of reasonable assumptions.

### Ecological/epidemiological background and modelling assumptions

The mosquito lifecycle is described as follows: After emerging, an adult female mosquito mates, blood-feeds, and lays eggs that progress through immature stages (eggs-larvae-pupae), eventually emerging as new adult females and males. Mosquitoes acquire *Plasmodium* infection after biting a human infected with malaria. The parasite is ingested in the form of *gametocytes* and progresses to the stage of *sporozoites*, which are present in the salivary glands after a certain time called the *Plasmodium* incubation period, which we neglect in this study. The endosymbiont *MB* is transmitted both vertically and horizontally (Herren et al 2020; Bukhari et al 2022; Nattoh et al 2021). Vertical transmission occurs when the endosymbiont is passed on to the progeny of an *MB*-infected adult female. It is perfect when all progeny are infected and imperfect when only a proportion $\beta _{v}$ of the progeny is infected. On the other hand, horizontal transmission occurs by mating between a wild mosquito and an *MB*-infected mosquito partner. The probability of transmission from an *MB*-infected male to a wild female is denoted by the male-to-female horizontal transmission rate $\beta _{h}^{mf}$, while the probability of transmission from an *MB*-infected female to a wild male $\beta _{h}^{fm}$ is called the female-to-male horizontal transmission rate. Mosquitoes infected with *Microsporidia MB* have the ability to block the transmission of *Plasmodium*. Specifically, if these mosquitoes bite a human infected with malaria and acquire the *Plasmodium* (through the multiple blood meals (Shaw et al 2020)), the parasite will not reach the *sporozoite* stage. The lab experiments suggest the *Plasmodium* transmission-blocking occurs either because of immune priming or because *MB* affects the mosquito metabolism (Herren et al 2020). Malaria is transmitted to humans by bite of a *Plasmodium*-infected mosquito, which transfers the *Plasmodium* parasite. Taking into account the mosquito life cycle, the transmission-blocking ability of *Microsporidia MB* infected mosquitoes, the malaria infection in humans and mosquitoes and the *Plasmodium* parasite transmission in humans, our eco-epidemiological model is based on the following main assumptions:

- Females mate once during their lifetime (Patinvoh and Susu 2014).
- Females always mate before blood-feeding. As a result, *Plasmodium*-infected mosquitoes cannot get the *MB*-infection.
- We neglect the average time for mating after emergence. So, female mosquitoes mate once they emerge.
- The sex (males, females) ratio in the immature stages is considered (1:1).

- We neglect the *Plasmodium* incubation period.
- Homogeneous distribution of wild and *MB*-infected mosquitoes: The probability of mating with an *MB*-infected partner is defined by the proportion of *MB*-infected mosquitoes.
- The *MB* infection is conserved throughout the lifespan of the mosquitoes.
- We consider the same death rates for wild and *MB*-infected mosquitoes, as lab experiments have not shown a significant effect on the fitness of *MB*-infected mosquitoes.
- Mosquitoes infected with both *MB* and *Plasmodium* do not contribute to malaria transmission, as the *Plasmodium* parasite do not reach the sporozoite stage.
- Constant human population.
- Neglected immunity after malaria infection.

Based on these assumptions, we define equations to represent the interactive dynamics of mosquitoes, *MB*, *Plasmodium* and humans.

### Model structure and equations

1 $$\begin{aligned} {\left\{ \begin{array}{ll} \dfrac{dF_{s}}{dt} &\, =\, b\left( 1-\dfrac{F_{T}}{K}\right) \left[ F_{1} + (1-\beta _{v})F_{2}\right] \left( 1 - \beta _{h}^{mf}\epsilon _{m}\dfrac{M_{m}}{M_{T}}\right) - \eta p^{hm}\dfrac{I_{h}}{N_{0}}F_{s} - d_{f}F_{s}; \\ \\ \dfrac{dF_{m}}{dt} &\, =\, \beta _{v}b\left( 1-\dfrac{F_{T}}{K}\right) F_{2} + b\left( 1-\dfrac{F_{T}}{K}\right) \left[ F_{1} + (1-\beta _{v})F_{2}\right] \beta _{h}^{mf}\epsilon _{m}\dfrac{M_{m}}{M_{T}} - \eta p^{hm}\dfrac{I_{h}}{N_{0}}F_{m} - d_{f}F_{m} + \alpha F_{mp} \\ \\ \dfrac{dF_{p}}{dt} &\, =\,\eta p^{hm}\dfrac{I_{h}}{N_{0}}F_{s} - d_{f}F_{p};\\ \\ \dfrac{dF_{mp}}{dt} &\, =\, \eta p^{hm}\dfrac{I_{h}}{N_{0}}F_{m} - \alpha F_{mp} - d_{f}F_{mp}; \\ \\ \dfrac{dM_{s}}{dt} &\, =\, b\left( 1-\dfrac{F_{T}}{K}\right) \left[ F_{1} + (1-\beta _{v})F_{2}\right] - \beta _{v}b\left( 1-\dfrac{F_{T}}{K}\right) F_{2}\beta _{h}^{fm}\epsilon _{w}\dfrac{M_{s}}{M_{T}} - d_{m}M_{s};\\ \\ \dfrac{dM_{m}}{dt} &\, =\, \beta _{v}b\left( 1-\dfrac{F_{T}}{K}\right) F_{2} + \beta _{v}b\left( 1-\dfrac{F_{T}}{K}\right) F_{2}\beta _{h}^{fm}\epsilon _{w}\dfrac{M_{s}}{M_{T}} - d_{m}M_{m};\\ \\ \dfrac{dI_{h}}{dt} &\, =\, \eta p^{mh}\dfrac{N_{0} - I_{h}}{N_{0}}F_{p} - \gamma _{h}I_{h}, \end{array}\right. } \end{aligned}$$

The mosquito population is categorized into mutually exclusive compartments based on *MB*-infection, *Plasmodium*-infection and the sex, as described in Table 1. This includes: wild females $(F_{s})$, wild males $(M_{s})$, *MB*-infected females $(F_{m})$ and *MB*-infected males $(M_{m})$, *Plasmodium*-infected females $(F_{p})$, *MB&Plasmodium*-infected females $(F_{mp})$. Furthermore, the number of humans infected with malaria is denoted by $I_{h}$ and the total human population is constant and indicated by $N_{0}$. The total female ($F_{T}$) and male populations ($M_{T}$) are denoted respectively by

$$\begin{aligned} F_{T} = F_{s} + F_{m} + F_{p} + F_{mp}, \quad \text {and} \quad M_{T} = M_{s} + M_{m}.\end{aligned}$$

It should be noted that $F_{p}$ represents the number of new *Plasmodium*-infections from the class ($F_{s}$) of wild female mosquitoes while $F_{mp}$ represents the number of new *Plasmodium*-infections from the class ($F_{m}$) of *MB*-infected mosquitoes.

For ease of writing, we also denote by $F_{1}$ (resp. $F_{2}$), the total number of female mosquitoes without (resp. with) *MB*. This is

$$\begin{aligned}F_{1}=F_{s} + F_{p}, \, \text {and} \, \, F_{2} = F_{m} + F_{mp}.\end{aligned}$$

The system of ordinary differential equations that governs the co-evolution of the mosquito population subject to *Microsporidia MB*, *Plasmodium* infections, and the human population threatened by malaria infection schematized in the flow diagram in Fig. 1 is given by the eco-epidemiological model (1). All the parameters involved in the model (1) are listed in Table 2. In a more comprehensive manner, the model (1) is constructed equation by equation along the following lines.

**Table 1:** Mosquito and human subpopulations

Notation	Definition
$F_{s}$	Wild female mosquitoes
$F_{m}$	*MB*-infected female mosquitoes
$F_{p}$	*Plasmodium*-infected females
$F_{mp}$	*MB&Plasmodium*-infected females
$M_{s}$	Wild male mosquitoes
$M_{m}$	*MB*-infected male mosquitoes
$I_{h}$	Malaria infected humans
$M_{T}$	Total male population
$F_{T}$	Total female population
$N_{0}$	Total human population
$F_{1} = F_{s} + F_{p}$	Total mosquitoes without *MB*
$F_{2} = F_{m} + F_{mp}$	Total mosquitoes with *MB*

The population of wild female mosquitoes ($F_{s}$) is increased over time by the emergence of new wild adult females, decreased by the new horizontally *MB*-infected female mosquitoes, the new *Plasmodium*-infected female mosquitoes and the natural female mosquito deaths. Let *b* be the half birth rate of a female mosquito, *K* the mosquito carrying capacity, and $\beta _{v}$ the vertical transmission rate. The number of new emerging wild female mosquitoes is equal to the sum of the offspring of wild adult female mosquitoes and the uninfected progeny of *MB*-infected adult females due to imperfect vertical transmission. Thus, recalling that $\beta _{v}$ is the efficiency or success of vertical transmission, $F_{1}$ the total female mosquitoes without *MB* and $F_{2}$, the total female mosquitoes with *MB*, the number of new emerging wild females is given by:

2 $$\begin{aligned} b\left( 1-\dfrac{F_{T}}{K}\right) \left[ F_{1} + (1-\beta _{v})F_{2}\right] . \end{aligned}$$

In addition, the number of new horizontally *MB*-infected females per unit of time is the number of newly emerged wild females that mate and successfully acquire the *MB*-infection. Therefore, it is the product of four quantities: the number of newly emerged wild females (2), the probability of mating with an *MB*-infected male partner ($M_{m}/M_{T}$), the relative attractiveness of *MB*-infected males to wild females $\epsilon _{m}$, and the successful horizontal transmission probability ($\beta _{h}^{mf}$) from male-to-female mosquitoes. Thus, it is expressed as follows:

3 $$\begin{aligned} b\left( 1-\dfrac{F_{T}}{K}\right) \left[ F_{1} + (1-\beta _{v})F_{2}\right] \beta _{h}^{mf}\epsilon _{m}\dfrac{M_{m}}{M_{T}}. \end{aligned}$$

Moreover, let $\eta$ denote the female mosquito biting rate and $p^{hm}$ the human-to-mosquito *Plasmodium* transmission efficiency. The number of new *Plasmodium*-infected female mosquitoes joining the $F_{p}$ class is:

4 $$\begin{aligned} \eta p^{hm}(I_{h}/N_{0})F_{s}. \end{aligned}$$

Finally, considering the natural female death rate $d_{f}$, the rate of change of $F_{s}$ is given by:

$$\begin{aligned} \begin{array}{rcl} \dfrac{dF_{s}}{dt} &\, =\, & b\left( 1-\dfrac{F_{T}}{K}\right) \left[ F_{1} + (1-\beta _{v})F_{2}\right] \\ \\ & - & b\left( 1-\dfrac{F_{T}}{K}\right) \left[ F_{1} + (1-\beta _{v})F_{2}\right] \beta _{h}^{mf}\epsilon _{m}\dfrac{M_{m}}{M_{T}} \\ \\ & - & \eta p^{hm}\dfrac{I_{h}}{N_{0}}F_{s} - d_{f}F_{s}. \end{array} \end{aligned}$$

The population of *MB*-infected female mosquitoes $F_{m}$ grows due to the number of newly emerged *MB*-infected females per unit of time,

5 $$\begin{aligned} \beta _{v}b\left( 1-\dfrac{F_{T}}{K}\right) F_{2}. \end{aligned}$$

and the number of newly horizontally *MB*-infected female mosquitoes (3). It diminishes due to the new *Plasmodium* infections,

6 $$\begin{aligned} \eta p^{hm}(I_{h}/N_{0})F_{m}. \end{aligned}$$

and the number of female mosquito deaths ($d_{f}F_{m}$). Moreover, thanks to the assumptions, if $\alpha$ is the rate of *Plasmodium* elimination within *MB&Plasmodium*-infected mosquitoes ($F_{mp}$), then the population of $F_{m}$ is further replenished by the term $\alpha F_{mp}$. Therefore, the rate of change of $F_{m}$ per unit of time is given by:

$$\begin{aligned} \begin{array}{rcl} \dfrac{dF_{m}}{dt} &\, =\, & \beta _{v}b\left( 1-\dfrac{F_{T}}{K}\right) F_{2} \\ \\ & + & b\left( 1-\dfrac{F_{T}}{K}\right) \left[ F_{1} + (1-\beta _{v})F_{2}\right] \beta _{h}^{mf}\epsilon _{m}\dfrac{M_{m}}{M_{T}} \\ \\ & - & \eta p^{hm}\dfrac{I_{h}}{N_{0}}F_{m} - d_{f}F_{m} + \alpha F_{mp}. \end{array} \end{aligned}$$

The population of mosquitoes infected with *Plasmodium* $F_{p}$ is increased due to new infections of susceptible mosquitoes by infected humans (4) and declines due to female deaths. So, the rate of change for $F_{p}$ per unit of time is:

$$\begin{aligned} \begin{array}{rcl} \dfrac{dF_{p}}{dt} = \eta p^{hm}\dfrac{I_{h}}{N_{0}}F_{s} - d_{f}F_{p}. \end{array} \end{aligned}$$

The population of *MB&Plasmodium*-infected mosquitoes $F_{mp}$ grows due to new infections by infected humans (6). It decreases due to the elimination of the *Plasmodium* infection, $\alpha F_{mp}$, and female deaths. Thus, the rate of change for $F_{mp}$ per unit of time is:

$$\begin{aligned} \begin{array}{rcl} \dfrac{dF_{mp}}{dt} = \eta p^{hm}\dfrac{I_{h}}{N_{0}}F_{m} - \alpha F_{mp} - d_{f}F_{mp}. \end{array} \end{aligned}$$

The population of wild male mosquitoes $M_{s}$ rises with the number of newly emerged wild males per unit of time, decreases with the newly horizontally *MB*-infected male mosquitoes, and is further reduced due to male mosquitoes deaths. Given the assumption of an equal sex ratio, the number of new emerging wild males and wild females are equal and defined in (2). In addition, using the conservation of the contacts during the mating process (Heffernan et al 2014; Podder and Gumel 2009), the number of new horizontally *MB*-infected male mosquitoes is equal to the number of emerging *MB*-females that have mated and successfully transfer the infection to wild male mosquitoes. This number can be given as a product of four quantities defined similarly as above. Namely: the number of newly emerging *MB*-infected females (5), the probability of mating with a wild male $(M_{s}/M_{T})$, the attractiveness of wild males to *MB*-infected female mosquitoes $\epsilon _{w}$, and the successful horizontal transmission probability ($\beta _{h}^{fm}$). Therefore, the number of new horizontally *MB*-infected male mosquitoes per unit of time is expressed by:

7 $$\begin{aligned} \beta _{v}b\left( 1-\dfrac{F_{T}}{K}\right) F_{2}\beta _{h}^{fm}\epsilon _{w}\dfrac{M_{s}}{M_{T}}. \end{aligned}$$

Finally, taking into account the male mosquito death rate $d_{m}$, the rate of change for $M_{s}$ per unit of time is:

$$\begin{aligned} \begin{array}{rcl} \dfrac{dM_{s}}{dt} &\, =\, & b\left( 1-\dfrac{F_{T}}{K}\right) \left[ F_{1} + (1-\beta _{v})F_{2}\right] \\ \\ & -& \beta _{v}b\left( 1-\dfrac{F_{T}}{K}\right) F_{2}\beta _{h}^{fm}\epsilon _{w}\dfrac{M_{s}}{M_{T}} - d_{m}M_{s}. \end{array} \end{aligned}$$

The population of *MB*-infected male mosquitoes $M_{m}$ increases with the number of newly emerged *MB*-infected males per unit of time given in (5) (thanks to the assumption of equal sex ratio), and the newly horizontally *MB*-infected males $new\_hor_{m}$ given in (7). It is reduced by the male mosquitoes deaths. As a result, the rate of change for $M_{m}$ per unit of time is:

$$\begin{aligned} \begin{array}{rcl} \dfrac{dM_{m}}{dt} &\, =\, & \beta _{v}b\left( 1-\dfrac{F_{T}}{K}\right) F_{2} \\ \\ & + & \beta _{v}b\left( 1-\dfrac{F_{T}}{K}\right) F_{2}\beta _{h}^{fm}\epsilon _{w}\dfrac{M_{s}}{M_{T}} - d_{m}M_{m}. \end{array} \end{aligned}$$

The population of humans infected with malaria $I_{h}$ is replenished due to new malaria infections, which are determined by the product of the mosquito biting rate $\eta$ times the mosquito-to-human *Plasmodium* transmission efficiency $p^{mh}$ times the *Plasmodium*-infected female mosquitoes $F_{p}$ multiplied by proportion of susceptible humans $((N_{0} - I_{h})/N_{0})$. Specifically, new malaria infections are estimated by $\eta p^{mh}((N_{0} - I_{h})/N_{0})F_{p}$. This population is reduced by the recoveries at rate $\gamma _{h}$. Hence, the rate of change for $I_{h}$ per unit of time is:

$$\begin{aligned} \begin{array}{rcl} \dfrac{dI_{h}}{dt}= & \eta p^{mh}\dfrac{N_{0} - I_{h}}{N_{0}}F_{p} - \gamma _{h}I_{h}. \end{array} \end{aligned}$$

This concludes the formulation of the model. The next section focuses on its mathematical analysis.

**Table 2:** Variable and parameters

Param	Definition	Value	References
*b*	Half female mosquito birth rate	0.09 (0.065$-$0.115)/day	(Maharaj 2003)
$d_{f}$	Female mosquito death rate	0.035 (0.02$-$0.05)/day	(Maharaj 2003)
$d_{m}$	Male mosquito death rate	0.05 (0.04$-$0.06)/day	(Maharaj 2003)
$1/\alpha$	Plasmodium elimination time in *MB*-infected mosquitoes	2–3/day	Assumed
$\eta$	Mosquito biting rate on a human	0.35 (0.2$-$0.5)/day	(Ruan et al 2008)
$p^{hm}$	*Plasmodium* transmission efficiency from humans to mosquito	0.5	(Ruan et al 2008)
$p^{mh}$	*Plasmodium* transmission efficiency from mosquito to humans	0.5	(Ruan et al 2008)
$\gamma _{h}$	Human recovering rate	0.01$-$0.05/day	(Ruan et al 2008)
$\beta _{v}$	Efficiency of vertical transmission	0.45–1	(Herren et al 2020)
$\beta _{h}^{fm}$	Efficiency of horizontal transmission from female to male	0.33 (0.2$-$0.6)	(Nattoh et al 2021)
$\beta _{h}^{mf}$	Efficiency of horizontal transmission from male to female	0.56 (0.2$-$0.6)	(Nattoh et al 2021)
$N_{0}$	Total human population	100 000	Assumed
*K*	Female mosquitoes carrying capacity	200 000	Estimated [1]
$\epsilon _{m}$	Attractiveness of *MB*-infected males for wild females	–	Assumed
$\epsilon _{w}$	Attractiveness of susceptible males for *MB*-infected females	–	Assumed
$T_{f}, \ T_{m}$	Equilibrium value of the total female and male mosquito populations, respectively	–	–
$t_{i}$	*Plasmodium* extrinsic incubation period	5–15 days	(Ruan et al 2008)

The carrying capacity is estimated assuming the mosquito to humans ratio of 2 (Ruan et al 2008)

## Mathematical and theoretical results

The mathematical analysis of System (1) consists of investigating its well-posedness, the existence and stability of ecological/epidemiological meaningful equilibrium points, the exhibition of different thresholds, as well as possible bifurcation types occurring at certain critical points.

### Theorem 1

(Well-posedness) The solution of System (1) appended with non-negative initial conditions is non-negative and bounded in ${\mathbb {R}}^{7}_{+}$. Moreover, System (1) is a dynamical system in the eco-epidemiological meaningful domain $\Omega$ defined by:

8 $$\begin{aligned} \Omega = \left\{ \begin{array}{l} \left( F_s, F_m, F_p, F_{mp}, M_s, M_m, I_h \right) \in {\mathbb {R}}^{7}_{+}, \\ \\ \, 0 \le F_s + F_{m} + F_p + F_{mp} \le T_{f}, \\ \\ \ 0 \le M_s + M_m \le \dfrac{d_{f}T_{f}}{d_{m}}, \, 0 \le I_h \le N_0 \end{array} \right\} \end{aligned}$$

where

$$\begin{aligned}T_{f} = K\left( 1 - \dfrac{d_{f}}{b}\right) \, \text {and} \; \, T_m = \dfrac{d_{f}T_{f}}{d_{m}};\end{aligned}$$

are the equilibrium values for the total population of female and male mosquitoes, respectively.

### Proof

The right-hand side of model (1) is locally Lipschitz on its definition domain. Therefore, any initial value problem of (1) has a unique solution. The positivity of the solutions of (1) is shown using the tangent argument by analyzing the flow on the boundary of ${\mathbb {R}}^{7}_{+}$. In other words, we ensure that the system does not allow any of his positive orbit to cross the boundary of ${\mathbb {R}}^{7}_{+}$. To do this, observe from (1) that if $F_{s} = 0$, then $dF_{s}/dt \ge 0$; if $F_{m} = 0$, then $dF_{m}/dt \ge 0$; if $F_{p} = 0$, then $dF_{p}/dt \ge 0$; if $F_{mp} = 0$, then $dF_{mp}/dt \ge 0$; if $M_{s} = 0$, then $dM_{s}/dt \ge 0$; if $M_{m} = 0$, then $dM_{m}/dt \ge 0$; if $I_{h} = 0$, then $dI_{h}/dt \ge 0$. This ensures the positivity for each of the state variables $F_{s}, \ F_{m}, \ F_{p}, \ F_{mp}, \ M_{s}, \ M_{m}, \ I_{h}$. Furthermore, we prove the invariance of $\Omega$ by analyzing the following system that govern the dynamics of the total female mosquito population ($F_{T}$) and the total male mosquito population ($M_{T}$),

$$\begin{aligned} \left\{ \begin{array}{rcl} \dfrac{dF_{T}}{dt} &\, =\, & (b-d_{f})F_{T}\left( 1 - \dfrac{F_{T}}{T_{f}}\right) . \\ \\ \dfrac{dM_{T}}{dt} &\, =\, & bF_{T}\left( 1 - \dfrac{F_{T}}{K}\right) - d_{m}M_{T}; \end{array} \right. \end{aligned}$$

Now, assume that the initial condition satisfies $F_{T0} \le T_{f}$ and $M_{T0} \le T_{m}$. Then, since $F_{T}$ follows a logistic growth equation, one can easily solve that equation and show that

$$\begin{aligned}F_{T}(t) \le T_{f} \ \text {for all time} \ t \ge 0;\end{aligned}$$

It then follows that:

$$\begin{aligned}\dfrac{dM_{T}}{dt} \le d_{f}T_{f} - d_{m}M_{T},\end{aligned}$$

from which the application of the comparison theorem for differential equations leads to

$$\begin{aligned} M_{T}(t) \le T_{m}, \ \text {for all time} \ t \ge 0.\end{aligned}$$

Finally, it is clear that if $I_{h} = N_{0}$, then $dI_{h}/dt < 0$, indicating that $I_{h}$ remains below $N_{0}$ together with its initial value. Therefore, we conclude that the domain $\Omega$ is invariant under the dynamics of System (1). $\square$

### Threshold parameters

The existence and stability analysis of the equilibrium points highlights three key threshold numbers that determine the long-term behavior of our system. We have:

1. *A threshold for extinction/persistence of MB-infected mosquitoes: *$R_{m}$ *or equivalently *$R_{1}$.
  We emphasize that there are three pathways of *MB*-infection:
  Vertical transmission mode from an already *MB*-infected female. The number of new *MB*-infected offspring is given by $\Gamma _{1} = (b/d_{f})\beta _{v}$.
  Vertical transmission from a female infected by a newly introduced *MB*-infected male. A male would infect $(T_{f}/d_{m}T_{m})\beta _{h}^{mf}\epsilon _{m}$ females during its lifetime. $T_{f}/T_{m}$ represents the number of female mosquitoes per male mosquito or equivalently the total number of female mosquitoes, a male mosquito will mate with per unit of time. Considering the birth rate *b*, given the equality $d_{f}T_{f} = d_{m}T_{m}$, the number of new *MB*-infected offspring resulting from this mode of transmission is given by,
    $$\begin{aligned} \begin{array}{lcl} \Gamma _{2} &\, =\,& b\dfrac{T_{f}}{d_{m}T_{m}}\beta _{h}^{mf}\epsilon _{m}\beta _{v} \\ \\ &\, =\, & \dfrac{b}{d_{f}}\beta _{v}\beta _{h}^{mf}\epsilon _{m}. \end{array} \end{aligned}$$
  Vertical transmission from a female, infected by an *MB*-infected male that has been infected by a newly introduced *MB*-infected female. Considering the unique mating occurrence for females, a female can infect $\beta _{h}^{fm}\epsilon _{w}$ males. It follows up from the previous bullet, that the number of new *MB*-infected offspring resulting from this mode of transmission is given by $\Gamma _{3} = (b/d_{f})\beta _{v}\beta _{h}^{mf}\epsilon _{m}\beta _{h}^{fm}\epsilon _{w}$.
  Combining the three threshold quantities above gives:
  $$\begin{aligned} \Gamma = \dfrac{b}{d_{f}}\left( \beta _{v} + \beta _{v}\epsilon _{m}\beta _{h}^{mf} + \beta _{v}\epsilon _{m}\beta _{h}^{mf}\epsilon _{w}\beta _{h}^{fm}\right) . \end{aligned}$$
  As a result, the ratio of new *MB*-infected offspring, associated with the three modes of transmissions: direct vertical, indirect (vertical and male-to-female) and indirect (vertical and female-to-male horizontal transmission), to the wild offspring of a wild female is given by:
  $$\begin{aligned} R_{m} = \beta _{v} + \beta _{v}\epsilon _{m}\beta _{h}^{mf} + \beta _{v}\epsilon _{m}\beta _{h}^{mf}\epsilon _{w}\beta _{h}^{fm}. \end{aligned}$$
  If $R_{m} > 1$, the prevalence of *MB*-infected mosquitoes tends to increase, leading to the persistence of the *MB*-infected mosquito population.
  So, in the case of imperfect vertical transmission (i.e. $\beta _{v} < 1$), a combination of vertical and horizontal transmission is necessary for the prevalence of *MB*-infected mosquitoes to increase (i.e. to ensure $R_{m} > 1$). It is important to remark that the effect of the female-to-male horizontal transmission pathway ($\Gamma _{3}$) is conditioned by twice mating for males, male-to-female horizontal transmission efficiency and vertical transmission efficiency. Also, the effect of male-to-female horizontal transmission ($\Gamma _{2}$) is conditioned by vertical transmission efficiency. Thus, direct vertical transmission has the biggest effect, followed by male-to-female and female-to-male horizontal transmission.
  This finding is consistent with the threshold presented in (Mfangnia et al 2023).
  For technical reasons, during the stability analysis of equilibria, the following equivalent threshold quantity $R_{1}$ will be used in place of $R_{m}$.
  $$\begin{aligned}R_{1} = \dfrac{\epsilon _{m}\beta _{v}\beta _{h}^{mf}(1+\beta _{h}^{fm}\epsilon _{w})}{1-\beta _{v}}.\end{aligned}$$
  We can easily observe that:
  $$\begin{aligned}(R_{m}< 1 \Leftrightarrow R_{1} < 1) \ \text {and} \ (R_{m}> 1 \Leftrightarrow R_{1} > 1). \end{aligned}$$

#### Remark 1

It is important to note that the attractiveness values $\epsilon _{m}$ and $\epsilon _{w}$ (Table 2), which increases the probability of mating with an *MB*-infected or wild mosquito, both have an upper limit, which is verified whenever $R_{2} < 1$, with

$$\begin{aligned}R_{2}= \dfrac{(1-\beta _{v})}{1+\beta _{v}\beta _{h}^{fm}\epsilon _{w}} R_{1}.\end{aligned}$$

Note that the threshold quantity $R_{2}$ always satisfies $R_{2} < R_{1}$ and was set during computation of equilibria to assess their existence.

2. A threshold indicating the extinction or persistence of malaria in the absence of *MB*-infected mosquitoes: $R_{0}$.
  $$\begin{aligned}R_{0} = \sqrt{\dfrac{\eta ^{2} p^{mh}p^{hm}T_{f}}{\gamma _{h}d_{f}N_{0}}}.\end{aligned}$$
  $R_{0}$ describes the average number of secondary cases a primary infected human will induce in a population of susceptible humans. It is calculated as the product of the total number of mosquitoes infected by the primary human case ($(\eta (T_{f}/N_{0})p^{hm})/\gamma _{h}$) and the total number of infectious bites ($\eta p^{mh}/d_{f}$).
3. A threshold indicating the extinction or persistence of malaria in the presence of *MB*-infected mosquitoes denoted by $R_{p}$ and given by
  $$\begin{aligned}R_{p} = R_{0}\sqrt{1-p}\end{aligned}$$
  where, for $R_{2}$ satisfying $R_{2}<1$, we have
  $$\begin{aligned}1 - p = \dfrac{(1+\beta _{v}\beta _{h}^{fm}\epsilon _{w})(1-R_{2})}{\beta _{v}(R_{1}+\beta _{h}^{fm}\epsilon _{w})}.\end{aligned}$$
  Clearly, the equilibrium prevalence of *MB*-infected mosquitoes in the total female population *p* defined above satisfies:
  $$\begin{aligned}p< 1 \ \text {whenever} \ R_{2} < 1.\end{aligned}$$
  Epidemiologically, $R_{p}$ gives the number of secondary cases a primary infected human will induce in a population of susceptible humans in the presence of *MB*-infected mosquitoes. We observe that $R_{p}$ decreases with an increasing prevalence of *MB*-infected mosquitoes. Thus, increasing the prevalence of *MB*-infected mosquitoes contributes to reducing malaria incidence.

### Equilibrium points

#### Definition 1

We say that an equilibrium of model (1) is disease-free when there are neither infected humans nor malaria-transmitting mosquitoes.

Using Definition 1, there are three possible disease-free equilibria and two endemic equilibria.

1. Disease-free with wild mosquitoes equilibrium $E^{00}$ (*that always exists*).
  This situation corresponds to malaria extinction in the absence of *MB*-infected mosquitoes. Specifically, all the mosquitoes are wild, and we have:
  $$\begin{aligned} {\left\{ \begin{array}{ll} F_{s}^{00} = T_{f}, \ F_{m}^{00} = \ F_{p}^{00} = \ F_{mp}^{00} = 0; \\ \\ M_{s}^{00} = T_{m}, \ M_{m}^{00} = \ I_{h}^{00} = 0. \end{array}\right. } \end{aligned}$$
2. Disease-free with complete *MB*-infection equilibrium $E^{01^{c}}$(*existing whenever *$\beta _{v} = 1$).
  In this scenario, the endosymbiont *MB* invades the mosquito population leading to the elimination of wild mosquitoes. Thus, there is no wild mosquito, no *Plasmodium*-infected mosquitoes, no malaria-infected humans and the equilibrium is defined by:
  $$\begin{aligned} {\left\{ \begin{array}{ll} F_{s}^{01^{c}} = 0; \ F_{m}^{01^{c}} = T_{f}; \ F_{p}^{01^{c}} = \ F_{mp}^{01^{c}} = 0; \\ \\ M_{s}^{01^{c}} = 0; \ M_{m}^{01^{c}} = T_{m}; \ I_{h}^{01^{c}} = 0. \end{array}\right. } \end{aligned}$$
3. Disease-free with wild and *MB*-infected mosquitoes equilibrium $E^{01}$(*existing whenever *$R_{1} > 1$ *and* $R_{2} < 1$). This scenario corresponds to the extinction of malaria in the presence of *MB*-infected mosquitoes. This equilibrium is defined as follows:
  9 $$\begin{aligned} {\left\{ \begin{array}{ll} F_{s}^{01} = \dfrac{T_{f}R_{p}^{2}}{R_{0}^{2}}; \ F_{m}^{01} = \dfrac{T_{f}(R_{0}^{2}-R_{p}^{2})}{R_{0}^{2}};\\ \\ F_{p}^{01} = 0; \ F_{mp}^{01} = 0; \ M_{s}^{01} = \dfrac{T_{m}}{R_{1}};\\ \\ M_{m}^{01} = T_{m}\left( 1-\dfrac{1}{R_{1}}\right) ; \ I_{h} = 0. \end{array}\right. } \end{aligned}$$
4. The endemic and *MB*-free equilibrium $E^{10}$ (*existing whenever *$R_{0} > 1$). This scenario corresponds to malaria persistence in the absence of *MB*-infected mosquitoes. In this situation, wild and *Plasmodium*-infected mosquitoes coexist, leading to an endemic malaria equilibrium where:
  10 $$\begin{aligned} {\left\{ \begin{array}{ll} F_{s}^{10} = \dfrac{T_{f}(\beta _{1}+d_{f}R_{0}^{2})}{R_{0}^{2}(\beta _{1}+d_{f})}; \ F_{m}^{10} = 0; \\ \\ F_{p}^{10} = \dfrac{\beta _{1}T_{f}(R_{0}^{2}-1)}{R_{0}^{2}(\beta _{1}+d_{f})}; \ F_{mp}^{10} = 0; \\ \\ M_{s}^{10} = T_{m}; \ \ M_{m}^{10} = 0; \\ \\ I_{h}^{10} = \dfrac{d_{f}N_{0}(R_{0}^{2}-1)}{d_{f}R_{0}^{2}+\beta _{1}}. \end{array}\right. } \end{aligned}$$
5. Complete coexistence equilibrium $E^{11}$ (whenever $R_{1} > 1$, $R_{2} < 1$ and $R_{p} > 1$).
  This scenario corresponds to the persistence of malaria in the presence of *MB*-infected mosquitoes. The equilibrium is given by:
  11 $$\begin{aligned} {\left\{ \begin{array}{ll} F_{s}^{11} =\dfrac{T_{f}(\beta _{1}+d_{f}R_{p}^{2})}{R_{0}^{2}(\beta _{1}+d_{f})}; \ F_{p}^{11} =\dfrac{\beta _{1}T_{f}(-1+R_{p}^{2})}{R_{0}^{2}(\beta _{1}+d_{f})}; \\ \\ F_{m}^{11} =\dfrac{(d_{f}+\alpha )(\beta _{1}+d_{f}R_{p}^{2})\tilde{F_{m}}}{(d_{f}+\alpha )(\beta _{1}+d_{f}R_{p}^{2})+\beta _{1}d_{f}(-1+R_{p}^{2})}; \\ \\ F_{mp}^{11} =\dfrac{\beta _{1}d_{f}(-1+R_{p}^{2})\tilde{F_{m}}}{(d_{f}+\alpha )(\beta _{1}+d_{f}R_{p}^{2})+\beta _{1}d_{f}(-1+R_{p}^{2})}; \\ \\ M_{m}^{11} = T_{m}\left( 1-\dfrac{1}{R_{1}}\right) ; \ M_{s}^{11} = \dfrac{T_{m}}{R_{1}}; \\ \\ \ I_{h}^{11} = \dfrac{d_{f}N_{0}(-1+R_{p}^{2})}{\beta _{1}+d_{f}R_{p}^{2}}; \end{array}\right. } \end{aligned}$$
  where,
  $$\begin{aligned}\tilde{F_{m}} = \dfrac{T_{f}(R_{0}^{2}-R_{p}^{2})}{R_{0}^{2}}.\end{aligned}$$

The long-term behaviour of model 1 is summarized in the stability result of equilibrium points below.

#### Theorem 2

(Local stability) The following statements hold.

- The *disease-free with wild mosquitoes equilibrium* $E^{00}$ is locally asymptotically stable whenever $R_{1} < 1$ and $R_{0} < 1$.
- The *endemic and MB-free equilibrium *$E^{10}$ is locally asymptotically stable whenever $R_{1} < 1$ and $R_{0} > 1$.
- The *disease-free with complete MB-infection equilibrium *$E^{01^{c}}$ exists and is locally asymptotically stable whenever $\beta _{v} = 1$.
- The *disease-free with wild and MB-infected mosquitoes* equilibrium $E^{01}$ is locally asymptotically stable whenever $R_{1} > 1$, $R_{2} < 1$ and $R_{p} < 1$.
- The *complete coexisting equilibrium *$E^{11}$ is locally asymptotically stable whenever $R_{1} > 1$, $R_{2} < 1$ and $R_{p} > 1$.

#### Remark 2

When $\beta _{v} = 1$, that is vertical transmission is perfect, then mosquito population replacement occurs and $E^{00}$ becomes $E^{10}$, which is always locally asymptotically stable.

**Table 3:** Equilibria stability

Equilibria	Existence condition	Stability condition
*Disease-free with wild mosquitoes * $E^{00}$	Always exists	$R_{1} < 1$ & $R_{0} < 1$
*The endemic and MB-free * $E^{10}$	$R_{0} > 1$	$R_{1} < 1$ & $R_{0} > 1$
*Disease-free with complete MB-infection * $E^{01^{c}}$	$\beta _{v} = 1$	$\beta _{v} = 1$
*Disease-free with wild and MB * $E^{01}$	$R_{1} > 1$ & $R_{2} < 1$	$R_{1} > 1$, $R_{2} < 1$ & $R_{p} < 1$
*Complete coexisting equilibrium * $E^{11}$	$R_{1} > 1$, $R_{2} < 1$ & $R_{p} > 1$	$R_{1} > 1$, $R_{2} < 1$ & $R_{p} > 1$

#### Proof

We denote by $\beta _{1} = \eta p^{hm}$. The linearized system (corresponding to System (1)) about a generic equilibrium of model (1)

$$\begin{aligned}E^{*} = (F_{s}^{*}, \ F_{m}^{*}, \ F_{p}^{*}, \ F_{mp}^{*}, \ M_{s}^{*}, \ M_{m}^{*}, \ I_{h}^{*});\end{aligned}$$

is given by:

$$\begin{aligned}\dfrac{dY}{dt}(t) = J_{0}Y(t),\end{aligned}$$

where,

$$Y(t)=(F_{s}(t), \ F_{m}(t), \ F_{p}(t), \ F_{mp}(t), \ M_{s}(t), \ M_{m}(t), \ I_{h}(t));$$

and $J_{0}$ is the Jacobian matrix with respect to *Y*(*t*) evaluated at the given equilibrium. In general, we get the following characteristic equation:

$$\begin{aligned}\psi (x) = (-b+d_{f}-x)(-d_{m}-x)\left( -d_{f}-\alpha -\dfrac{\beta _{1}I_{h}^{*}}{N_{0}} - x\right) \psi _{1}(x)\psi _{2}(x);\end{aligned}$$

where,

12 $$\begin{aligned} \begin{array}{rcl} \psi _{1}(x) &\, =\,& \left( d_{f}(\beta _{v}-1-\beta _{v}\Delta _{1})-x\right) \left( -d_{m}-\theta _{2}\beta _{h}^{fm}\dfrac{\epsilon _{w}}{T_{m}}-x\right) \\ & -& \theta _{1}\beta _{h}^{mf}\dfrac{\epsilon _{m}}{T_{m}}d_{f}\beta _{v}(1+\Delta _{2}); \end{array} \end{aligned}$$

and

13 $$\begin{aligned} \begin{array}{rcl} \psi _{2}(x) &\, =\,& \left( -d_{f}-\dfrac{\beta _{1}I_{h}^{*}}{N_{0}}-x\right) \left( -\gamma _{h}-\dfrac{\eta p^{mh}F_{p}^{*}}{N_{0}}-x\right) \\ & -& \dfrac{\eta p^{mh}(N_{0}-I_{h}^{*})\beta _{1}F_{s}^{*}}{N_{0}^{2}}; \end{array} \end{aligned}$$

with

14 $$\begin{aligned} \left\{ \begin{array}{rcl} \theta _{1} &\, =\,& d_{f}(F_{s}^{*}+F_{p}^{*}+(1-\beta _{v})(F_{m}^{*}+F_{mp}^{*})), \\ \\ \theta _{2} &\, =\,& \beta _{v}d_{f}(F_{m}^{*}+F_{mp}^{*})), \ \Delta _{1} = \beta _{h}^{mf}\dfrac{\epsilon _{m}M_{m}^{*}}{T_{m}}, \\ \\ \Delta _{2} &\, =\,& \beta _{h}^{fm}\dfrac{\epsilon _{w}M_{s}^{*}}{T_{m}}. \end{array} \right. \end{aligned}$$

Then, to conclude the local asymptotic stability of an equilibrium of System 1, we analyse the sign of the real part of the roots of $\psi _{1}$ and $\psi _{2}$.

1. Local asymptotic stability of the *disease-free with wild mosquitoes equilibrium *$E^{00}$;
  In this case, we get
  $$\begin{aligned} \begin{array}{rcl} \psi _{1}(x) &\, =\,& x^{2}+(d_{m}+(1-\beta _{v})d_{f})x \\ \\ & +& (1-\beta _{v})d_{f}d_{m}\left( 1-R_{1}\right) ; \end{array} \end{aligned}$$
  and
  $$\begin{aligned} \psi _{2}(x) = x^{2}+(d_{f}+\gamma _{h})x+d_{f}\gamma _{h}(1-R_{0}^{2}). \end{aligned}$$
  The eigenvalues of $\psi _{1}(x)$ and $\psi _{2}(x)$ have negative real parts by Routh–Hurwitz criteria whenever $R_{1} < 1$ and $R_{0} < 1$. Then, we conclude the local asymptotic stability when $R_{1} < 1$ and $R_{0} < 1$.
2. Local asymptotic stability of the *endemic and MB-free equilibrium *$E^{10}$;
  In this case, we get:
  $$\begin{aligned} \begin{array}{rcl} \psi _{1}(x) &\, =\,& x^{2}+(d_{m}+(1-\beta _{v})d_{f})x \\ \\ & +& (1-\beta _{v})d_{f}d_{m}\left( 1-R_{1}\right) ; \end{array} \end{aligned}$$
  and
  $$\begin{aligned} \psi _{2}(x) = x^{2}+\left( \dfrac{d_{f}R_{0}^{2}}{A}+\gamma _{h}A\right) x+d_{f}\gamma _{h}\left( R_{0}^{2}-1\right) ; \end{aligned}$$
  where $$A =(d_{f}R_{0}^{2}+\beta _{1})/(d_{f}+\beta _{1})$$.
  The eigenvalues of $\psi _{1}(x)$ and $\psi _{2}(x)$ have negative real parts by Routh-Hurwitz criteria whenever $R_{1} < 1$ and $R_{0} > 1$. Then, we conclude the local asymptotic stability when $R_{1} < 1$ and $R_{0} > 1$.
3. Local asymptotic stability of the *disease-free with complete MB-infection equilibrium *$E^{01^{c}}$;
  We get
  $$\begin{aligned}\psi _{1}(x) = (-d_{f}\beta _{h}^{fm}\epsilon _{m}-x)(-d_{m}(1+\beta _{h}^{fm}\epsilon _{w})-x);\end{aligned}$$
  and
  $$\begin{aligned}\psi _{2}(x) = (-d_{f}-x)(-\gamma _{h}-x).\end{aligned}$$
  Then, we conclude the local asymptotic stability whenever it exists ($\beta _{v} = 1$).
4. Local asymptotic stability of the *disease-free with wild and MB-infected mosquitoes* equilibrium $E^{01}$;
  After computations,
  $$\begin{aligned} \psi _{1}(x) = x^{2}-tr(x)x+d_{m}d_{f}(1-\beta _{v})(R_{1}-1); \end{aligned}$$
  where,
  $$\begin{aligned} \begin{array}{rcl} tr(x) &\, =\,& -\dfrac{d_{m}R_{1}(1+\beta _{h}^{fm}\epsilon _{w})}{R_{1}+\beta _{h}^{fm}\epsilon _{w}}-\dfrac{d_{f}(1-\beta _{v})\epsilon _{w}\beta _{h}^{fm}}{1+\beta _{h}^{fm}\epsilon _{w}} \\ \\ & -& \beta _{v}d_{f}\beta _{h}^{mf}\epsilon _{m} < 0. \end{array} \end{aligned}$$
  In addition,
  $$\begin{aligned} \psi _{2}(x) = x^{2}+(d_{f}+\gamma _{h})x+d_{f}\gamma _{h}(1-R_{p}^{2}). \end{aligned}$$
  The eigenvalues of $\psi _{1}(x)$ and $\psi _{2}(x)$ have negative real parts by Routh-Hurwitz criteria whenever $R_{1} > 1$, $R_{2} < 1$ and $R_{p} < 1$. Then, we conclude the local asymptotic stability when $R_{1} > 1$, $R_{2} < 1$ and $R_{p} < 1$.
5. Local asymptotic stability of the *complete coexisting equilibrium *$E^{11}$;
  In this case, $\psi _{1}(x)$ has the same expression as above:
  $$\begin{aligned} \psi _{1}(x) = x^{2}-tr(x)x+d_{m}d_{f}(1-\beta _{v})(R_{1}-1); \end{aligned}$$
  and
  $$\begin{aligned}\psi _{2}(x) = x^{2}+\left( \dfrac{d_{f}R_{p}^{2}}{A}+\gamma _{h}A\right) x+d_{f}\gamma _{h}\left( R_{p}^{2}-1\right) ;\end{aligned}$$
  where $$A =(d_{f}R_{0}^{2}+\beta _{1})/(d_{f}+\beta _{1})$$.
  The eigenvalues of $\psi _{1}(x)$ and $\psi _{2}(x)$ have negative real parts by Routh-Hurwitz criteria whenever $R_{1} > 1$ and $R_{p} > 1$. Then, we conclude the local asymptotic stability when $R_{1} > 1$, $R_{2} < 1$ and $R_{p} > 1$.

This concludes the proof of Theorem 2.

$$\square$$

In summary, *MB*-infected mosquitoes go extinct when $R_{1} < 1$ and persist when $R_{1} > 1$. When $R_{1} > 1$, the presence of *MB*-infected mosquitoes results in three additional scenarios: a disease-free equilibrium associated with perfect vertical transmission, and both a disease-free equilibrium and malaria persistence equilibrium associated with imperfect vertical transmission. For imperfect vertical transmission, the stability of these equilibria is determined by the new threshold $R_{p}$, where $R_{p} < 1$ indicates malaria extinction and $R_{p} > 1$ indicates malaria persistence. Therefore, in the presence of *MB*-infected mosquitoes, it is advantageous to achieve $R_{p} < 1$. Table 3 summarizes the equilibria and stability conditions.

#### Theorem 3

(Global stability) The following statements hold.

- The *disease-free with wild mosquitoes equilibrium* $E^{00}$ is globally asymptotically stable in $\Omega$, whenever $R_{1} < 1$ and $R_{0} < 1$.
- The *endemic and MB-free equilibrium *$E^{10}$ is globally asymptotically stable in the interior of $\Omega$, whenever $R_{1} < 1$ and $R_{0} > 1$.
- The *disease-free with complete MB-infection equilibrium *$E^{01^{c}}$ exists and is globally asymptotically stable in $\Omega \backslash \{E^{00}\}$, when $\beta _{v} = 1$.
- The *disease-free with wild and MB-infected mosquitoes* equilibrium $E^{01}$ is globally asymptotically stable in $\Omega \backslash \{E^{00}\}$, whenever $R_{1} > 1$, $R_{2} < 1$ and $R_{p} < 1$.
- The *complete coexisting equilibrium *$E^{11}$ is globally asymptotically stable in the interior of $\Omega$, when $R_{1} > 1$, $R_{2} > 1$ and $R_{p} > 1$.

#### Proof

For the proof, it is important to observe that by summing the equations describing the time variation of $F_{s}, \ F_{m}, \ F_{p},$ and $F_{mp}$, we get:

15 $$\begin{aligned} \dfrac{dF_{T}}{dt} = bF_{T}\left( 1-\dfrac{F_{T}}{K}\right) -d_{f}F_{T} = \dfrac{bT_{f}}{K}F_{T}\left( 1-\dfrac{F_{T}}{T_{f}}\right) . \end{aligned}$$

We observe from equation (15), that $F_{T}$ follows a logistic growth and we deduce that:

$$\begin{aligned}\lim _{t\rightarrow +\infty } F_{T} = T_{f}.\end{aligned}$$

Thus, at $+\infty$, the total male mosquito population verifies $dM_{T}/dt = d_{f}T_{f} - d_{m}M_{T}$, and we easily deduce that:

$$\begin{aligned}\lim _{t\rightarrow +\infty } M_{T} = \dfrac{d_{f}T_{f}}{d_{m}} = T_{m}.\end{aligned}$$

**Table 4:** Some Convergence criteria

Convergence	Criteria	Ref
$\displaystyle \lim _{t \rightarrow +\infty } F_{m} = \lim _{t \rightarrow +\infty } F_{mp} = \lim _{t \rightarrow +\infty } M_{m} = 0$	$R_{1} < 1$	(17)
$\displaystyle \lim _{t \rightarrow +\infty } F_{2} = T_{f}(R_{0}^{2}-R_{p}^{2})/R_{0}^{2}$, $\displaystyle \lim _{t \rightarrow +\infty } F_{1} = T_{f}R_{p}^{2}/R_{0}^{2}$		
$\displaystyle \lim _{t \rightarrow +\infty } M_{s} = T_{m}/R_{1}$ and $\displaystyle \lim _{t \rightarrow +\infty }$ $M_{m} = T_{m}(1-1/R_{1})$	$R_{1} > 1$	(19)
$\displaystyle \lim _{t \rightarrow +\infty } F_{p} = \lim _{t \rightarrow +\infty } I_{h} = \lim _{t \rightarrow +\infty } F_{mp} = 0$	($R_{1} < 1$ & $R_{0} < 1$)	
	or ($R_{1} > 1$ & $R_{p} < 1$)	(23)
$$\displaystyle \lim _{t \rightarrow +\infty } F_{s} = \lim _{t \rightarrow +\infty } F_{p} = \lim _{t \rightarrow +\infty } F_{mp} = \lim _{t \rightarrow +\infty } M_{s} = 0$$		
$\displaystyle \lim _{t \rightarrow +\infty } F_{m} = T_{f}, \ \lim _{t \rightarrow +\infty } M_{m} = T_{m}$	$\beta _{v} = 1$	(27)
$\displaystyle \lim _{t \rightarrow +\infty } F_{p} = \dfrac{\beta _{1}T_{f}(R_{0}^{2}-1)}{R_{0}^{2}(\beta _{1}+d_{f})} \ \text {and} \ \lim _{t \rightarrow +\infty } I_{h} = \dfrac{d_{f}N_{0}(R_{0}^{2}-1)}{d_{f}R_{0}^{2}+\beta _{1}}$	($R_{1} < 1 \ \& \ R_{0} > 1$)	(25)
$\displaystyle \lim _{t \rightarrow +\infty } F_{p} = \dfrac{\beta _{1}T_{f}(R_{p}^{2}-1)}{R_{0}^{2}(\beta _{1}+d_{f})} \ \text {and} \ \lim _{t \rightarrow +\infty } I_{h} = \dfrac{d_{f}N_{0}(R_{p}^{2}-1)}{d_{f}R_{p}^{2}+\beta _{1}}$	($R_{1}> 1 \ \& \ R_{p} > 1$)	(26)

Let us consider the two categories of mosquitoes: those without *MB* ($$F_{1} = F_{s} + F_{p}$$) and those with *MB* ($$F_{2} = F_{m} + F_{mp})$$. We can represent our model as a system of two submodels: the first submodel captures the dynamics of $$F_{2} \ \text {and} \ M_{m}$$,

$$\begin{aligned} \left\{ \begin{array}{l} \dfrac{dF_{2}}{dt} = b\left( 1-\dfrac{F_{T}}{K}\right) \left( \beta _{v}F_{2} + (T_{f}-\beta _{v}F_{2})\beta _{h}^{mf}\epsilon _{m}\dfrac{M_{m}}{M_{T}}\right) - d_{f}F_{2} \\ \\ \dfrac{dM_{m}}{dt} = \beta _{v}b\left( 1-\dfrac{F_{T}}{K}\right) F_{2} \left( 1 + \beta _{h}^{fm}\epsilon _{w}\dfrac{M_{T} - M_{m}}{M_{T}} - d_{m}M_{m} \right) . \end{array} \right. \end{aligned}$$

while the second submodel describes the dynamics of $F_{p}, \ \text {and} \ I_{h}$:

$$\begin{aligned} \left\{ \begin{array}{l} \dfrac{dF_{p}}{dt} = \eta p^{hm}\dfrac{I_{h}}{N_{0}}(F_{1} - F_{p}) - d_{f}F_{p}; (F_{1} = F_{T} - F_{2})\\ \\ \dfrac{dI_{h}}{dt} = \eta p^{mh}\dfrac{N_{0} - I_{h}}{N_{0}}F_{p} - \gamma _{h}I_{h}, \end{array} \right. \end{aligned}$$

From the decoupling of our model (1) described above, we observe that to establish the stability of its equilibria, it is sufficient to prove the convergence, as outlined in Table 4, which we demonstrate in the following lines.

Given that $\displaystyle \lim _{t \rightarrow +\infty } F_{T} = T_{f}$ and $\displaystyle \lim _{t \rightarrow +\infty } M_{T}$, as established above, $F_{2}$ and $M_{m}$ satisfy the following limiting system:

16 $$\begin{aligned} \left\{ \begin{array}{l} \dfrac{dF_{2}}{dt} = \beta _{v}d_{f}F_{2} + d_{f}(T_{f}-\beta _{v}F_{2})\beta _{h}^{mf}\epsilon _{m}\dfrac{M_{m}}{T_{m}} - d_{f}F_{2} \\ \\ \dfrac{dM_{m}}{dt} = \beta _{v}d_{f}F_{2}\left( 1 + \beta _{h}^{fm}\epsilon _{w}\left( 1-\dfrac{M_{m}}{T_{m}}\right) \right) - d_{m}M_{m} \end{array} \right. \end{aligned}$$

This system admits two equilibria: a zero equilibria

$$\begin{aligned}(F_{2} = 0, \ M_{m} = 0)\end{aligned}$$

and a non-zero equilibria, when $R_{1} > 1$, given by:

$$\begin{aligned}\left( F_{2}^{*} = T_{f}\left( 1 - \dfrac{R_{p}^{2}}{R_{0}^{2}}\right) , \ M_{m}^{*} = T_{m}\left( 1 - \dfrac{1}{R_{1}}\right) \right) \end{aligned}$$

In the following, we demonstrate the global asymptotical stability for these two equilibria. Starting with the zero equilibria, we consider the following Lyapunov candidate function:

$$\begin{aligned}V_{1} = F_{2} + \beta _{h}^{mf}\epsilon _{m}M_{m}.\end{aligned}$$

Using the equality $d_{f}T_{f} = d_{m}T_{m}$, the derivative of $V_1$ is given by:

$$\begin{aligned} \begin{array}{rcl} \dfrac{dV_{1}}{dt} &\, =\,& \beta _{v}d_{f}F_{2} + d_{f}(T_{f}-\beta _{v}F_{2})\beta _{h}^{mf}\epsilon _{m}\dfrac{M_{m}}{T_{m}} - d_{f}F_{2} \\ & +& \beta _{h}^{mf}\epsilon _{m} \left( \beta _{v}d_{f}F_{2}\left( 1 + \beta _{h}^{fm}\epsilon _{w}\left( 1-\dfrac{M_{m}}{T_{m}}\right) \right) - d_{m}M_{m} \right) ; \\ \\ &\, =\,& d_{f}F_{2}\left[ -1 + \beta _{v} + \beta _{h}^{mf}\epsilon _{m}\beta _{v}\left( 1 + \epsilon _{w}\beta _{h}^{fm}\left( 1-\dfrac{M_{m}}{T_{m}}\right) \right) \right] \\ & -& \beta _{v}d_{f}F_{2}\beta _{h}^{mf}\epsilon _{m}\dfrac{M_{m}}{T_{m}}. \end{array} \end{aligned}$$

Then, using the fact that $M_{m}\le T_{m}$, we have:

$$\begin{aligned}dV_{1}/dt \le d_{f}F_{2}(1-\beta _{v})(-1 + R_{1}) - \beta _{v}d_{f}F_{2}\beta _{h}^{mf}\epsilon _{m}\dfrac{M_{m}}{T_{m}}.\end{aligned}$$

Thus, $dV_{1}/dt \le 0$ whenever $R_{1} < 1$. Also, $dV_{1}/dt = 0$ if and only if $F_{2} = 0$. It can be verified that the largest invariant set in which $dV_{1}/dt = 0$ is the singleton $\left\{ (F_{2} = 0, \ M_{m} = 0)\right\}$. Thus, it follows from the LaSalle invariance principle, that, when $R_{1} < 1$,

$$\begin{aligned}\lim _{t \rightarrow +\infty } F_{2} = \lim _{t \rightarrow +\infty } M_{m} = 0,\end{aligned}$$

or equivalently, when $R_{1} < 1$, we obtain:

17 $$\begin{aligned} \lim _{t \rightarrow +\infty } F_{m} = \lim _{t \rightarrow +\infty } F_{mp} = \lim _{t \rightarrow +\infty } M_{m} = 0. \end{aligned}$$

We now proceed with proving the global asymptotic stability of the non-zero equilibrium $(F_{2}^{*}, \ M_{m}^{*})$ using the *Dulac’s criterion*. In so doing, consider the following Dulac candidate function:

$$\begin{aligned}H(F_{2}, \ M_{m}) = \dfrac{1}{F_{2}M_{m}}.\end{aligned}$$

Then, *F* denotes the right hand side of (16), we have:

$$\begin{aligned} \begin{array}{rcl} \nabla (H.F) &\, =\,& \dfrac{\partial }{\partial F_{2}}\left( \dfrac{1}{F_{2}M_{m}}\dfrac{dF_{2}}{dt}\right) + \dfrac{\partial }{\partial M_{m}}\left( \dfrac{1}{F_{2}M_{m}}\dfrac{dM_{m}}{dt}\right) ; \\ \\ &\, =\,& \dfrac{\partial }{\partial F_{2}}\left( \dfrac{\beta _{v}d_{f}}{M_{m}} + \dfrac{d_{f}T_{f}\beta _{h}^{mf}\epsilon _{m}}{T_{m}F_{2}} - \dfrac{\beta _{v}d_{f}\beta _{h}^{mf}\epsilon _{m}}{T_{m}} - \dfrac{d_{f}}{M_{m}}\right) \\ & +& \dfrac{\partial }{\partial M_{m}}\left( \dfrac{\beta _{v}d_{f}}{M_{m}} + \beta _{v}d_{f}\beta _{h}^{fm}\epsilon _{w}\left( \dfrac{1}{M_{m}} - \dfrac{1}{T_{m}}\right) - \dfrac{d_{m}}{F_{2}}\right) ;\\ \\ &\, =\,& - \dfrac{d_{m}\beta _{h}^{mf}\epsilon _{m}}{F_{2}^{2}} - \dfrac{\beta _{v}d_{f}(1 + \beta _{h}^{fm}\epsilon _{w})}{M_{m}^{2}}. \end{array} \end{aligned}$$

$\nabla (H.F)$ has the same sign everywhere in our domain $\Omega$ (8). Thus, there is no closed orbit and the only equilibrium is globally stable. We conclude:

18 $$\begin{aligned} \left\{ \begin{array}{l} \displaystyle \lim _{t \rightarrow +\infty } F_{2} = T_{f}\left( 1-\dfrac{R_{p}^{2}}{R_{0}^{2}}\right) ; \\ \displaystyle \lim _{t \rightarrow +\infty } M_{m} = T_{m}\left( 1 - \dfrac{1}{R_{1}}\right) . \end{array} \right. \end{aligned}$$

From (18), and the established relationships, $F_{T} = F_{1} + F_{2}$, $\lim _{t \rightarrow +\infty } F_{T} = T_{f}, \ \text {and} \ \lim _{t \rightarrow +\infty } M_{T} = T_{m}$, we deduce that:

19 $$\begin{aligned} \begin{array}{ll} \left\{ \begin{array}{l} \displaystyle \lim _{t \rightarrow +} F_{2} = T_{f}(R_{0}^{2}-R_{p}^{2})/R_{0}^{2}, \\ \\ \displaystyle \lim _{t \rightarrow +\infty } F_{1} = T_{f}R_{p}^{2}/R_{0}^{2}, \\ \\ \displaystyle \lim _{t \rightarrow +\infty } M_{s} = T_{m}/R_{1} \ \text {and} \\ \\ \displaystyle \lim _{t \rightarrow +\infty } M_{m} = T_{m}(1-1/R_{1}) \end{array} \right.&\end{array} \end{aligned}$$

By establishing the convergence results in (17) and (19), we have shown that there is global asymptotic convergence to an *MB*-free state when $R_{1} > 1$, and *MB* persists when $R_{1} > 1$, for any non-zero initial conditions. Next, to conclude on the global asymptotic behaviour for the *Plasmodium*-infected mosquitoes and malaria-infected humans, we consider the second submodel:

20 $$\begin{aligned} \left\{ \begin{array}{l} \dfrac{dF_{p}}{dt} = \eta p^{hm}\dfrac{I_{h}}{N_{0}}(F_{1} - F_{p}) - d_{f}F_{p};\\ \\ \dfrac{dI_{h}}{dt} = \eta p^{mh}\dfrac{N_{0} - I_{h}}{N_{0}}F_{p} - \gamma _{h}I_{h}, \end{array} \right. \end{aligned}$$

We already know the global asymptotic behaviour of $F_{1}$, which is:

$$\begin{aligned} \left\{ \begin{array}{l} \lim _{t \rightarrow +\infty } F_{1} = T_{f}, \ \text {when} \ R_{1} < 1 \\ \\ \lim _{t \rightarrow +\infty } F_{1} = T_{f}R_{p}^{2}/R_{0}^{2}, \ \text {when} \ R_{1} > 1 \end{array} \right. 
\end{aligned}$$

Thus, when $R_{1} < 1$, (20) is asymptotically equivalent to:

21 $$\begin{aligned} \left\{ \begin{array}{l} \dfrac{dF_{p}}{dt} = \eta p^{hm}\dfrac{I_{h}}{N_{0}}(T_{f} - F_{p}) - d_{f}F_{p};\\ \\ \dfrac{dI_{h}}{dt} = \eta p^{mh}\dfrac{N_{0} - I_{h}}{N_{0}}F_{p} - \gamma _{h}I_{h}, \end{array} \right. \end{aligned}$$

and when $R_{1} > 1$, (20) is asymptotically equivalent to:

22 $$\begin{aligned} \left\{ \begin{array}{l} \dfrac{dF_{p}}{dt} = \eta p^{hm}\dfrac{I_{h}}{N_{0}}(\dfrac{T_{f}R_{p}^{2}}{R_{0}^{2}} - F_{p}) - d_{f}F_{p};\\ \\ \dfrac{dI_{h}}{dt} = \eta p^{mh}\dfrac{N_{0} - I_{h}}{N_{0}}F_{p} - \gamma _{h}I_{h}, \end{array} \right. \end{aligned}$$

Let us consider the zero equilibrium $(F_{p} = 0, \ I_{h} = 0)$ and the following Lyapunov candidate function:

$$\begin{aligned}V_{2} = F_{p} + \dfrac{d_{f}}{\eta p^{mh}}I_{h}.\end{aligned}$$

When $R_{1} < 1$, the time derivative of $V_2$ is:

$$\begin{aligned} \begin{array}{rcl} \dfrac{dV_{2}}{dt} &\, =\,& \eta p^{hm}\dfrac{I_{h}}{N_{0}}T_{f} - d_{f}F_{p} \\ & +& \dfrac{d_{f}}{\eta p^{mh}}\left[ \eta p^{mh}\dfrac{N_{0} - I_{h}}{N_{0}}F_{p} - \gamma _{h}I_{h}\right] ; \\ \\ &\, =\,& \dfrac{d_{f}\gamma _{h}}{\eta p^{mh}}I_{h}\left( -1 + R_{0}^{2}\right) - \dfrac{d_{f}F_{p}I_{h}}{N_{0}}.\\ \end{array} \end{aligned}$$

Thus, if ($$R_{1} < 1$$ and $$R_{0} < 1$$) then $$dV_{2}/dt \le 0$$. Similarly, from (22), it is easy to show that if ($$R_{1} < 1$$ and $$R_{p} < 1)$$, then $$dV_{2}/dt \le 0$$. We conclude:

23 $$\begin{aligned} \left\{ \begin{array}{l} \text {whenever} \ (R_{1}< 1 \ \& \ R_{0}< 1) \\ \text {or} \ (R_{1} > 1 \ \& \ R_{p} < 1), \\ \displaystyle \lim _{t \rightarrow +\infty } F_{p} = \lim _{t \rightarrow +\infty } I_{h} = \lim _{t \rightarrow +\infty } F_{mp} = 0 \ \end{array} \right. \end{aligned}$$

When ($R_{1} < 1 \ \& \ R_{0} > 1$), we consider the limit system (21). It is clear that the unique non-trivial and locally asymptotically stable equilibrium of (2.2) is given by:

$$\begin{aligned}\left( \dfrac{\beta _{1}T_{f}(R_{0}^{2}-1)}{R_{0}^{2}(\beta _{1}+d_{f})}, \dfrac{d_{f}N_{0}(R_{0}^{2}-1)}{d_{f}R_{0}^{2}+\beta _{1}}\right) . \end{aligned}$$

Now, according to Theorem 3.8.5 in (Li 2018), to conclude the global asymptotic stability of the non-trivial equilibria of (2.2), it remains to establish the strong monotonicity of System (2.2), as well as the strict sub-linearity of the functions *f* and *g* defined by $(f(F_{p}, \ I_{h}), \ g(F_{p}, \ I_{h})) = \left( dF_{p}/dt, \ dI_{h}/dt\right)$.

In so doing, let *J* be the Jacobian matrix associated with System (2.2)0. Then

24 $$\begin{aligned} J =\begin{bmatrix} -\dfrac{\beta _{1}I_{h}}{N_{0}}-d_{f} & \dfrac{\beta _{1}T_{f}}{N_{0}}\\ \\ \eta p^{mh}\left( 1-\dfrac{I_{h}}{N_{0}}\right) & -\dfrac{\eta p^{mh}F_{p}}{N_{0}}-\gamma _{h} \end{bmatrix} \end{aligned}$$

Clearly, *J* is irreducible and Metzler matrix in $\Omega$. Thus, System (2.2) is strictly monotone. For the strict sub-linearity of the right-hand side of (2.2) denoted by $(f(F_{p}, \ I_{h}), \ g(F_{p}, \ I_{h}))$, suppose that $0< \lambda < 1$, ($F_{p} > 0$, $I_{h} > 0$), then

$$\begin{aligned} \begin{array}{r c l} f(\lambda F_{p}, \ \lambda I_{h}) &\, =\,& \beta _{1}\dfrac{\lambda I_{h}}{N_{0}}(T_{f}-\lambda F_{p}) - d_{f}\lambda F_{p} \\ & >& \lambda \left( \beta _{1}\dfrac{I_{h}}{N_{0}}(T_{f}-F_{p}) - d_{f}F_{p}\right) \\ &\, =\,& \lambda f(F_{p}, \ I_{h}). \end{array} \end{aligned}$$

Similarly, we can show $g(\lambda F_{p}, \ \lambda I_{h}) > \lambda g(F_{p}, \ I_{h})$, and conclude the sub-linearity of *f* and *g*. In addition, the endemic equilibrium is the only locally asymptotic stable equilibrium when $R_{0} > 1$. Thanks to Theorem 3.8.5 in (Li 2018), we conclude its global asymptotic stability when $R_{0} > 1$.

Thus, we conclude that whenever ($R_{1} < 1 \ \& \ R_{0} > 1$),

25 $$\begin{aligned} \begin{array}{rl} \left\{ \begin{array}{l} \displaystyle \lim _{t \rightarrow +\infty } F_{p} = \dfrac{\beta _{1}T_{f}(R_{0}^{2}-1)}{R_{0}^{2}(\beta _{1}+d_{f})}, \\ \\ \displaystyle \lim _{t \rightarrow +\infty } I_{h} = \dfrac{d_{f}N_{0}(R_{0}^{2}-1)}{d_{f}R_{0}^{2}+\beta _{1}}, \end{array} \right. \end{array} \end{aligned}$$

Similarly, we can prove that if ($R_{1}> 1 \ \& \ R_{p} > 1$), then

26 $$\begin{aligned} \begin{array}{l} \left\{ \begin{array}{l} \displaystyle \lim _{t \rightarrow +\infty } F_{p} = \dfrac{\beta _{1}T_{f}(R_{p}^{2}-1)}{R_{0}^{2}(\beta _{1}+d_{f})} \\ \\ \displaystyle \lim _{t \rightarrow +\infty } I_{h} = \dfrac{d_{f}N_{0}(R_{p}^{2}-1)}{d_{f}R_{p}^{2}+\beta _{1}} \end{array} \right. \end{array} \end{aligned}$$

Furthermore, when $\beta _{v} = 1$ and $t\rightarrow + \infty$,

$$\begin{aligned}\dfrac{dF_{1}}{dt} = -d_{f}F_{1}\beta _{h}^{mf}\epsilon _{m}\dfrac{M_{s}}{T_{m}} \le 0\end{aligned}$$

Thus $F_{1}$ is a viable Lyapunov function when $\beta _{v} = 1$. Also, $dF_{1}/dt = 0$ if and only if $F_{1} = 0$ or $M_{s} = 0$. It is easy to observe that the largest invariant set where $dF_{1}/dt = 0$ is the singleton $\left\{ (F_{1} = 0, \ M_{s} = 0)\right\}$. Thus, it follows from the Lyapunov invariance principle, that $\displaystyle \lim _{t \rightarrow +\infty } F_{1} = \lim _{t \rightarrow +\infty } M_{s} = 0$, when $\beta _{v} = 1$. As a result,

27 $$\begin{aligned} \left\{ \begin{array}{l} \displaystyle \lim _{t \rightarrow +\infty } F_{s} = \lim _{t \rightarrow +\infty } F_{p} = \lim _{t \rightarrow +\infty } F_{mp} = \lim _{t \rightarrow +\infty } M_{s} = 0 \\ \\ \displaystyle \lim _{t \rightarrow +\infty } F_{m} = T_{f}, \ \lim _{t \rightarrow +\infty } M_{m} = T_{m}. \end{array} \right. \end{aligned}$$

Finally, thanks to the boundedness of solutions and local stability of the equilibria, we conclude that:

- Based on (17) and (23), $E^{00}$ is globally attractive and thus globally asymptotically stable (GAS) in $\Omega$ when $R_{1} < 1$ and $R_{0} < 1$.
- Based on (17) and (25), $E^{10}$ is globally attractive, and thus GAS in the interior of $\Omega$ when $R_{1} < 1$ and $R_{0} > 1$.
- Based on (27), $E^{01^{c}}$ is globally attractive, and thus GAS in $\Omega \backslash \{E^{00}\}$ when $\beta _{v} = 1$.
- Based on (23) and (19), $E^{01}$ is globally attractive, and thus GAS in $\Omega \backslash \{E^{00}\}$ when $R_{1} > 1$, $R_{2} < 1$ and $R_{p} < 1$.
- Based on (19) and (26), $E^{11}$ is globally attractive, and thus GAS in the interior of $\Omega$ when $R_{1} > 1$, $R_{2} < 1$ and $R_{p} > 1$.

$$\square$$

**Fig. 2:**
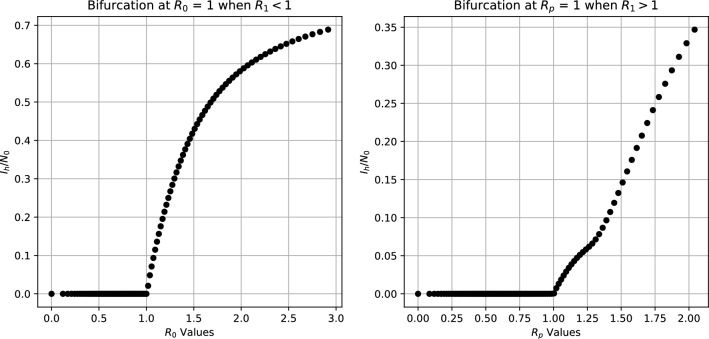
Forward bifurcation at $R_{0} = 1$ when $R_{1} < 1$ and at $R_{p} = 1$, when $R_{1} > 1$

#### Theorem 4

(Bifurcation result) System (1) presents:

(i) A two forward transcritical bifurcations: the first one at $R_{0} = 1$, when $R_{1} < 1$; and the second at $R_{p} = 1$, when $R_{1} > 1$.
(ii) A forward transcritical bifurcation at $R_{1} < 1$.

#### Proof

We have the following bifurcation phenomena:

(i) The forward transcritical bifurcation at $R_{0} = 1$ whenever $R_{1} < 1$ follows from Theorems 2 and 3. Indeed, whenever $R_{1} < 1$, the *disease-free with wild mosquito equilibrium *$E^{00}$ changes from stable ($R_{0} < 1$) to unstable ($R_{0} > 1$), while the *endemic and MB-free equilibrium *$E^{10}$ becomes positive from $E^{00}$ and asymptotically stable. Similarly, whenever $R_{1} > 1$, the *disease-free with wild and MB-infected mosquitoes* equilibrium $E^{01}$ changes from stable ($R_{p} < 1$) to unstable ($R_{p} > 1$), while the *complete coexisting equilibrium *$E^{11}$ becomes positive from $E^{01}$ and asymptotically stable.
(ii) In this case, an *MB*-free equilibrium (either $E^{00}$ {if $R_{0} < 1$} or $E^{10}$ {if $R_{0} > 1$}) changes from stable [$R_{1} < 1$] to unstable [$R_{1} > 1$]. While, an equilibria with *MB* (either $E^{01}$ {if $R_{p} < 1$} or $E^{11}$ {if $R_{p} > 1$}) appear and is stable ($R_{1} > 1$). It is worth noting that when $R_{1} > 1$, the *disease-free with wild mosquitoes equilibrium *$E^{00}$ give rise to the *disease-free with wild and MB-infected mosquitoes* equilibrium $E^{01}$. On the other hand, considering an initial $R_{0} > 1$, the outcome in terms of malaria spread (either elimination, which is associated with ($R_{p} < 1$ and $E^{01}$) or malaria persistence, which is associated with ($R_{p} > 1$ and $E^{11}$) will depend on the prevalence *p* of *MB* in female mosquitoes as $R_{p} = R_{0}\sqrt{1-p}$.

$$\square$$

**Fig. 3:**
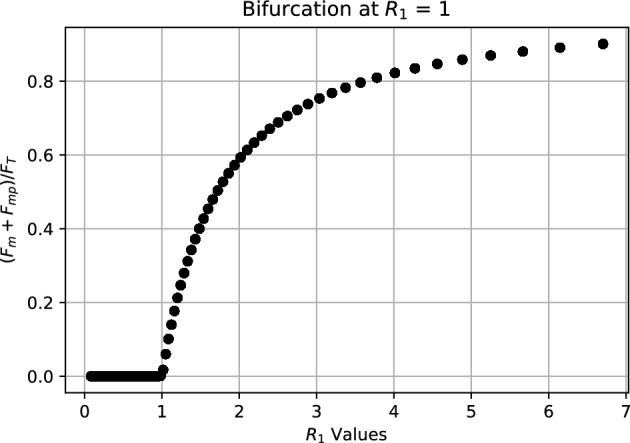
Forward bifurcation at $R_{1} = 1$

It is important to observe that the stable equilibrium when $R_{1} > 1$ depends on the malaria incidence in presence of *MB*-infected mosquitoes, measured by $R_{p}$, which is determined by the prevalence of *MB*-infected mosquitoes and malaria incidence in absence of *MB*-infected mosquitoes, measured by $R_{0}$. The typical bifurcation diagrams near the bifurcation points are illustrated in Figs. 2 and 3, respectively.

## Interpretation of the results

In this section, we provide an interpretation of the mathematical results. In the absence of *MB*-infected mosquitoes, we have two scenarios: a malaria-free state and a malaria-endemic state. The stability of this malaria-endemic equilibrium is ensured whenever $R_{0} > 1$, leading to a number of infected humans given by

28 $$\begin{aligned} I_{h1} = \dfrac{d_{f}N_{0}(R_{0}^{2}-1)}{d_{f}R_{0}^{2}+\beta _{1}}. \end{aligned}$$

In the presence of *MB*-infected mosquitoes, we have three possible long-term scenarios: a malaria-free state associated with complete *MB*-infection, a malaria-free state associated with coexisting wild and *MB*-infected mosquitoes and a malaria-endemic state where both *MB*-infected and wild mosquitoes coexist. The situation of all mosquitoes infected by symbionts can be achieved only when the vertical transmission is perfect ($\beta _{v} = 1$). Regarding the remaining scenarios, we can assess ranges of parameters complying with either an *MB*-free state or an *MB* &wild-coexistence state by evaluating the threshold $R_{1}$.

$$\begin{aligned}R_{1} = \dfrac{\epsilon _{m}\beta _{v}\beta _{h}^{mf}(1+\beta _{h}^{fm}\epsilon _{w})}{1-\beta _{v}}.\end{aligned}$$

This is done in the following subsection.

**Fig. 4:**
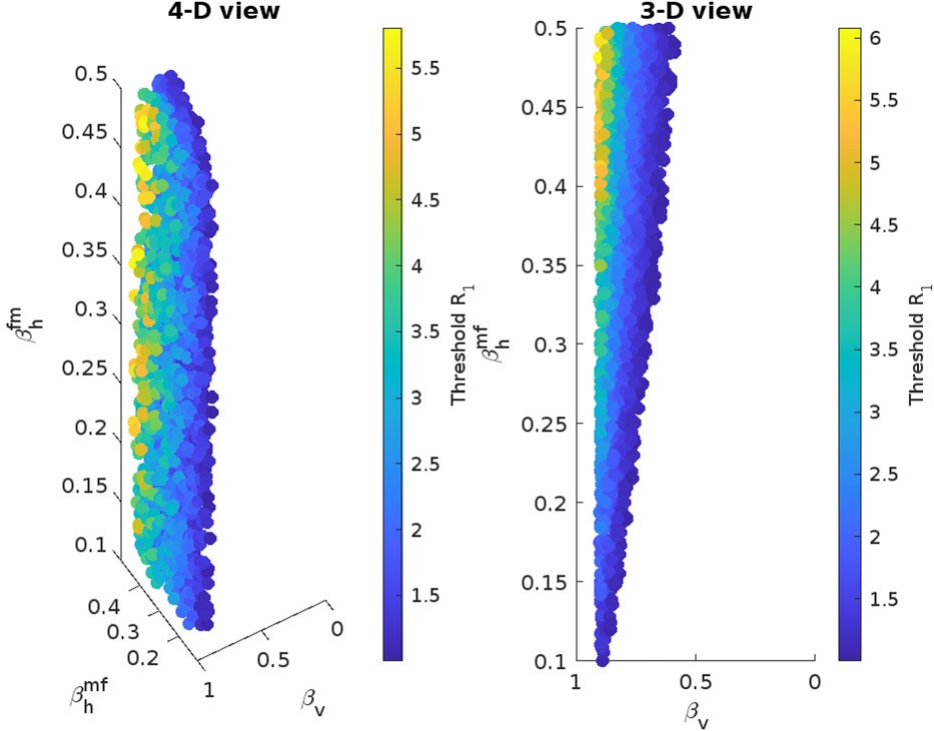
Fourth Dimensional plot of $\beta _{v}$, $\beta _{h}^{fm}$ and $\beta _{h}^{mf}$ values satisfying $R_{1} > 1$

### Effect of *MB* transmission rates on *MB* prevalence

Assuming the same attractiveness for *MB*-infected and wild males, meaning $\epsilon _{m} = \epsilon _{w} = 1$, and the efficiencies of horizontal transmission from male to female and female to a male lying in the range $[0-0.5]$, the variation of the threshold $R_{1}$ is depicted in Fig. 4.

**Fig. 5:**
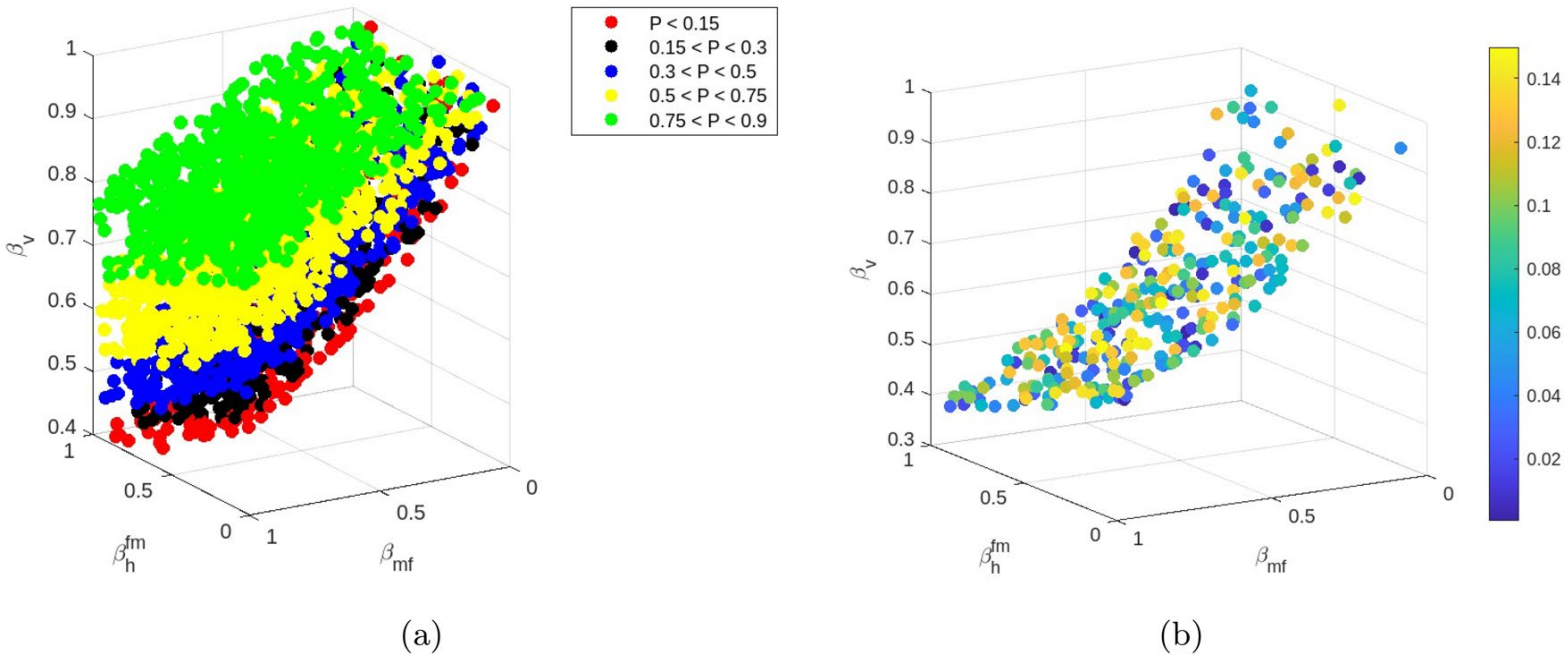
**a** Distribution of the prevalence *P* of *MB*-infected mosquitoes according to the horizontal and vertical transmission efficiencies $\beta _h^{fm}$, $\beta _h^{fm}$ and $\beta _{v}$. **b** Zoom on the efficiencies leading to a prevalence lower than 15%

The numerical analysis of the variation of $R_{1}$ (Fig. 4) with respect to the efficiencies $\beta _{v}$, $\beta _{h}^{fm}$ and $\beta _{h}^{mf}$ shows that for maximal values of horizontal transmission efficiencies in the range $0 - 0.5$, and the vertical transmission efficiency lower than 0.5, the condition $R_{1} < 1$ is verified and the *MB*-infected mosquitoes will go to extinction. In the alternative scenario, where $R_{1} > 1$, we evaluate the variation of the prevalence of *Microsporidia* in the mosquito population,

$$\begin{aligned}p = \dfrac{(R_{1}-1)}{T_{m}+T_{f}}\left( \dfrac{T_{f}}{\beta _{v}(R_{1}+\beta _{h}^{fm}\epsilon _{w})} + \dfrac{T_{m}}{R_{1}}\right) .\end{aligned}$$

There is quite a wide range of values of $\beta _{h}^{mf}$, $\beta _{h}^{fm}$ and $\beta _{v}$ complying with a prevalence of *MB*-infected mosquitoes lower than 15 % as it has been reported from field experiments (Herren et al 2020), presented in Table 5 and depicted in Fig. 5.

**Table 5:** Range of transmission efficiencies complying with a prevalence lower than 15%

$\beta _{v}$	$\beta _{h}^{mf}$	$\beta _{h}^{fm}$	Prevalence
0.17	0.31	0.85	0.12
0.46	0.48	0.64	0.10
0.51	0.34	0.65	0.14

Considering the average of vertical transmission efficiency, 0.72 (0.45–1 (Herren et al 2020)) and horizontal transmission efficiencies, around 20–50%, which values are consistent with those given in Table 5, we can also infer that the scenario of the coexistence of *MB* and wild mosquitoes is happening in nature. Since *Microsporidia MB* has been identified as a potential bio-based agent control for use to reduce malaria transmission, we analyze how the presence of *Microsporidia* affects malaria dynamics in the next subsection.

### Effect of *MB* infection on malaria incidence

In the presence and stability of the symbiont, meaning $R_{1} > 1$, and considering imperfect vertical transmission, there are two additional scenarios in the malaria dynamics: a second malaria-free state stable when $R_{p} < 1$ and a second malaria-endemic state stable when $R_{p} > 1$.

$$\begin{aligned}R_{p} = R_{0}\sqrt{1-p}.\end{aligned}$$

Since the prevalence of *MB*-infected mosquitoes in the female mosquito population is $p <1$, it is clear that $R_{p}< R_{0}$. From the expression of $R_{p}$, we deduce that the presence of symbiont contributes to reducing the basic reproduction number $R_{0}$ and to controlling malaria disease. Moreover, the number of infected humans at the second endemic state is given by:

29 $$\begin{aligned} I_{h2}=\dfrac{d_{f}N_{0}(-1+R_{p}^{2})}{\beta _{1}+d_{f}R_{p}^{2}}. \end{aligned}$$

By comparing the number of malaria-infected humans in the absence of *MB* (28) and the number of malaria-infected humans in the presence of *MB* (29), the percentage of malaria reduction achieved when the second malaria-endemic equilibrium is stable, is:

30 $$\begin{aligned} \left( 1-\dfrac{I_{h2}}{I_{h1}}\right) (\%) = \dfrac{p_{f}R_{0}^{2}(\beta _{1}+d_{f})}{(\beta _{1}+d_{f}R_{0}^{2}(1-p))(R_{0}^{2}-1)} (\%). \end{aligned}$$

We deduce from (30), that the percentage of malaria elimination achieved with a given prevalence p of *Microsporidia* in the mosquito population strongly depends on the initial basic reproduction number $R_{0}$. In other words, the percentage of malaria elimination achieved with a given prevalence p of *Microsporidia* in the mosquito population depends on the level of malaria transmission in a particular area and should be assessed accordingly. In the following lines, we estimate the contribution of the presence of *MB*-infected mosquitoes on malaria incidence reduction.

**Fig. 6:**
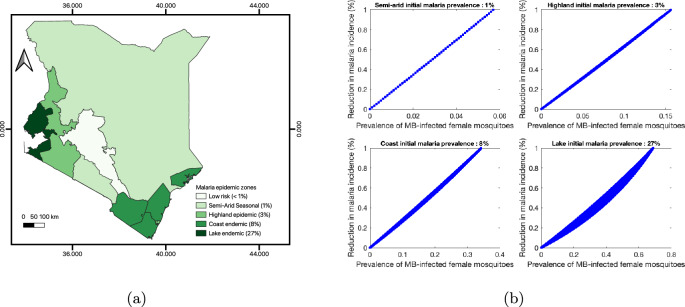
**a** Malaria prevalence in Kenya as presented in (Haileselassie et al 2023). **b** Malaria incidence reduction plotted against the prevalence of *Microsporidia MB* in mosquitoes

Let us consider the malaria prevalence distribution in Kenya, as shown in Fig. 6a.

We recall that according to our model formulation, the initial malaria prevalence can be obtained using the formula:

$$\begin{aligned} p_{I} = (I_{h1}/N_{0}) =d_{f}(R_{0}^{2}-1)/(d_{f}R_{0}^{2}+\beta _{1}). \end{aligned}$$

Then, for each estimated real malaria prevalence, we compute the corresponding basic reproduction number

31 $$\begin{aligned} R_{0}^{2} = (d_{f}+p_{I}\eta p^{hm})/(d_{f}(1-p_{I})), \end{aligned}$$

as resumed in Table 6.

**Table 6:** Prevalence and associated basic reproduction number considering formula (31) and the parameters in Table (2)

Prevalence	0.01	0.03	0.08	0.27
$R_{0}$	1.06	1.18	1.52	3.21

Finally, we estimate the percentage achieved in malaria reduction by simulating the expression (30) over a wide range of mosquito bite rate ($\eta \in [0.2, \ 0.5]$) and female mosquito death rate ($d_{f} \in [0.02, \ 0.05]$), for each initial malaria prevalence described in Fig. 6a. The corresponding graph is presented in Fig. 6b. We conclude, based on Fig. 6b, that:

- Malaria eradication can be achieved in the highland region with a 15% prevalence of *MB*-infected mosquitoes;
- Malaria eradication can be achieved on the coast region with 40% prevalence of *MB*-infected mosquitoes;
- Malaria eradication can be achieved in the lake region with 70% prevalence of *MB*-infected mosquitoes.

## Malaria dynamics with extrinsic incubation period

The model presented in (1) did not account for the extrinsic incubation period in the mosquito population. This could be done in two different ways. Firstly and directly by adding a new class of latently infected mosquitoes in the dynamics of mosquito population, or indirectly by introducing a delay in the equation of malaria-infected humans. For the sake of simplicity and to facilitate comparison with the model without inclusion of the incubation period, we adopt the latter approach. Therefore, if $t_{i}>0$ is the extrinsic incubation period and $e^{-d_{f}t_{i}}$ is the corresponding survival probability during the extrinsic incubation period, then the dynamics of individuals infected with malaria becomes:

$$\begin{aligned}\dfrac{dI_{h}}{dt} = \eta p^{mh}\dfrac{N_{0} - I_{h}}{N_{0}}F_{p}(t - t_{i})e^{-d_{f}t_{i}} - \gamma _{h}I_{h}.\end{aligned}$$

In this new model which takes into account the extrinsic incubation period, there are few changes compared with the model without explicit incubation period. The initial condition for the population of mosquitoes infected with *Plasmodium* becomes:

$$\begin{aligned}F_{p}(\theta ) = \phi (\theta ), \ \theta \ \in [-t_{i}, \ 0];\end{aligned}$$

Also, the threshold for the extinction or persistence of *MB*-infected mosquitoes $R_{1}$ remains the same, while the thresholds for malaria extinction or persistence ($R_{0}$ in the absence of *MB* and $R_{p}$ in the presence of *MB* are multiplied by $\sqrt{e^{-d_{f}t_{i}}}$). That is, for *p* defined as in Sect. 3.1, we have:

$$\begin{aligned}R_{0}^{eip} = R_{0}\sqrt{e^{-d_{f}t_{i}}}; \ R_{p}^{eip} = R_{0}^{eip}\sqrt{1-p}.\end{aligned}$$

We make the following observations:

- The equilibria of the model with extrinsic incubation period are given in Sect. 3.2, where $R_{0}$ is replaced by $R_{0}^{eip}$ and $R_{p}$ replaced by $R_{p}^{eip}$.
- The local stability of the equilibria as stated in Theorem 2 remains true when $R_{0}$ is replaced by $R_{0}^{eip}$ and $R_{p}$ by $R_{p}^{eip}$.
- The same global stability results for equilibria stated in Theorem 3 hold true when $R_{0}$ is replaced by $R_{0}^{eip}$ and $R_{p}^{eip}$ in place of $R_{p}$.

The proofs of these assertions are provided shortly.

### Proof

For the local asymptotic stability of the equilibria, the characteristic polynomial at the equilibrium point $E^{*} = (F_{s}^{*}, \ F_{m}^{*}, \ F_{p}^{*}, \ F_{mp}^{*}, \ M_{s}^{*}, \ M_{m}^{*}, \ I_{h}^{*})$ keeps the same form as in the proof of Theorem 2. That is:

$$\begin{aligned}\psi (x) = (-b+d_{f}-x)(-d_{m}-x)\left( -d_{f}-\alpha -\dfrac{\beta _{1}I_{h}^{*}}{N_{0}} - x\right) \psi _{1}(x)\psi _{2}(x);\end{aligned}$$

where, $\psi _{1}$ is defined as in (12) and $\psi _{2}$ becomes:

$$\begin{aligned} \begin{array}{rcl} \psi _{2}(x) &\, =\,& \left( -d_{f}-\dfrac{\beta _{1}I_{h}^{*}}{N_{0}}-x\right) \left( -\gamma _{h}-\dfrac{\eta p^{mh}e^{-d_{f}t_{i}}F_{p}^{*}}{N_{0}}-x\right) \\ & -& \dfrac{\eta p^{mh}(N_{0}-I_{h}^{*})e^{-d_{f}t_{i}}e^{-xt_{i}}\beta _{1}F_{s}^{*}}{N_{0}^{2}}. \end{array} \end{aligned}$$

Given that $\psi _{1}$ is the same as in (12), the necessary conditions for the roots of $\psi _{1}$ to have negative real parts are as follows: $E^{00}$ ($R_{1} < 1$), $E^{10}$ ($R_{1} < 1$), $E^{01^{c}}$ ($\beta _{v} = 1$), $E^{01}$ ($R_{1} < 1$ and $R_{2} < 1$) and $E^{11}$ ($R_{1} < 1$ and $R_{2} < 1$).

Table 7 below gives the expression for $\psi _{2}(x)$ for each equilibrium. In the case of $E^{00}$ and $E^{01}$, $\psi _{2}(x)$ takes the generic form:

$$\begin{aligned}j_{1}(x) = x^{2} + (b + c)x + bc(1 - r_{1}^{2}e^{-t_{i}x});\end{aligned}$$

whereas in case of $E^{10}$ and $E^{11}$, $\psi _{2}(x)$ takes the generic form:

$$\begin{aligned}j_{2}(x) = x^{2} + (\dfrac{br_{2}^{2}}{a} + ca)x + bc(r_{2}^{2} - e^{-t_{i}x});\end{aligned}$$

where *a*, *b*, *c* are positive numbers,  $$r_{1} = R_{0}^{eip}$$ for $$E^{00}$$, $$r_{1} = R_{p}^{eip}$$ for $$E^{01}$$, $$r_{2} = R_{0}^{eip}$$ for $$E^{10}$$ and $$r_{2} = R_{p}^{eip}$$ for $$E^{11}$$. It is enough to show that real parts of the roots of $$j_{1}$$ (resp. $$j_{2}$$) are negative under the condition $$r_{1} < 1$$ (resp. $$ r_2 > 1$$). We shall provide the proof for $$j_{1}(x)$$ and that for $$j_{2}(x)$$ follows in a similar way.

**Table 7:** Table summarizing the values of $\psi _{2}(x)$ for various equilibria

Eq.$^{1}$	Value of $\psi _{2}(x)$
$E^{00}$	$\psi _{2}(x) = x^{2}+(d_{f}+\gamma _{h})x+d_{f}\gamma _{h}(1-{R_{0}^{eip}}^{2}e^{-t_{i}x})$
$E^{10}$	$\psi _{2}(x) = x^{2}+\left( \dfrac{d_{f}{R_{0}^{eip}}^{2}}{A}+\gamma _{h}A\right) x+d_{f}\gamma _{h}\left( {R_{0}^{eip}}^{2}-e^{-t_{i}x}\right) ^{2}$
$E^{01^{c}}$	$\psi _{2}(x) = (-d_{f}-x)(-\gamma _{h}-x)$
$E^{01}$	$\psi _{2}(x) = x^{2}+(d_{f}+\gamma _{h})x+d_{f}\gamma _{h}(1-{R_{p}^{eip}}^{2}e^{-t_{i}x})$
$E^{11}$	$\psi _{2}(x) = x^{2}+\left( \dfrac{d_{f}{R_{p}^{eip}}{2}}{A}+\gamma _{h}A\right) x+d_{f}\gamma _{h}\left( {R_{p}^{eip}}^{2}-e^{-t_{i}x}\right)$

Equilibria

$$A =(d_{f}R_{0}^{2}+\beta _{1})/(d_{f}+\beta _{1})$$

It is straightforward that $j_{1}'(x) > 0$ for $x \in [0, \ +\infty ]$, $j_{1}(0) = c(1-r_{1}^{2})$ and $j_{1}(+\infty ) = + \infty$ and we discuss the sign of the real parts of the eigenvalues depending on the value of $r_{1}^{2}$ ($r_{1} < 1$ or $r_{1} > 1$).

If $r_{1} > 1$, $j_{1}(0) < 0$. Therefore, $j_{1}(x)$ has at least a positive root by continuity.

If $r_{1} < 1$ and $t_{i} = 0$, then the roots of

$$\begin{aligned}x^{2} + (b + c)x + bc(1 - r_{1}^{2}) = 0;\end{aligned}$$

have negative real parts by Routh-Hurwitz criteria. For $r_{1} < 1$ and $t_{i} > 0$, let us assume the existence of a root $w=iz, \ z > 0$ such that $j_{1}(w) = 0$. Then, *z* verify:

$$\begin{aligned}-z^{2} + i(b + c) z + bc = bcr_{1}^{2}(\cos (t_{i}z)-i\sin (t_{i}z)).\end{aligned}$$

Thus,

32 $$\begin{aligned} -z^{2} + bc = bcr_{1}^{2}\cos (t_{i}z) \ \text {and} \ (b + c)z = -bcr_{1}^{2}\sin (t_{i}z). \end{aligned}$$

After squaring the left and right sides of the equation in (32), $z \in {\mathbb {R}}^{+}$ is solution of

$$\begin{aligned}z^{4}+(b^{2}+c^{2})z^{2}+b^{2}c^{2}(1-r_{1}^{4}) = 0.\end{aligned}$$

Since $r_{1} < 1$, we conclude that there is no such $z \in {\mathbb {R}}^{+}$. Therefore, $j_{1}(x)$ has no purely imaginary root. It follows from Corollary 2.4 in (Ruan and Wei 2003) that all roots of $j_{1}(x)$ have negative real parts. $\square$

The global asymptotic stability of the equilibria results stated in Theorem 3 still hold true when $R_{0}$ is replaced by $R_{0}^{eip}$ and $R_{p}$ is replaced by $R_{p}^{eip}$.

### Proof

This proof is similar to that of Theorem 3. For this reason, we shall prove only the GAS of $E^{00}$. It is enough to establish a relation similar to (23) when $R_{0}$ is replaced by $R_{0}^{eip}$ and $R_{p}$ by $R_{p}^{eip}$. We consider the following Lyapunov function:

$$\begin{aligned}V_{2}^{1} = F_{p} + \dfrac{d_{f}e^{d_{f}t_{i}}}{\eta p^{mh}}I_{h} + d_{f}\int _{t-t_{i}}^{t}F_{p}(u)du.\end{aligned}$$

Then, its derivative, following System (21) is:

$$\begin{aligned} \begin{array}{rcl} \dfrac{dV_{2}^{1}}{dt} &\, =\,& \eta p^{hm}\dfrac{I_{h}}{N_{0}}T_{f} - d_{f}F_{p} + d_{f}(F_{p} - F_{p}(t - t_{i})) \\ & +& \dfrac{d_{f}e^{d_{f}t_{i}}}{\eta p^{mh}}\left[ \eta p^{mh}\dfrac{N_{0} - I_{h}}{N_{0}}F_{p}(t-t_{i})e^{-d_{f}t_{i}} - \gamma _{h}I_{h}\right] ; \\ \\ &\, =\,& \dfrac{d_{f}\gamma _{h}e^{d_{f}t_{i}}}{\eta p^{mh}}I_{h}\left( -1 + R_{0}^{2}\right) - \dfrac{d_{f}F_{p}(t-t_{i})I_{h}}{N_{0}}.\\ \end{array} \end{aligned}$$

Applying the same reasoning as in (23), we deduce that:

$$\begin{aligned} \left\{ \begin{array}{l} \displaystyle \lim _{t \rightarrow +\infty } F_{p} = \lim _{t \rightarrow +\infty } I_{h} = \lim _{t \rightarrow +\infty } F_{mp} = 0 \\ \text {when} \ (R_{1}< 1 \ \& \ R_{0}^{eip}< 1) \ \text {or} \ (R_{1} > 1 \ \& \ R_{p}^{eip} < 1). \end{array} \right. \end{aligned}$$

$$\square$$

**Fig. 7:**
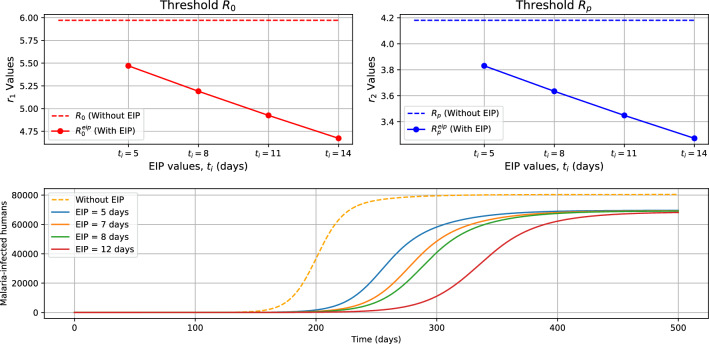
Malaria dynamics with and without extrinsic incubation period (EIP). From left to right, the variation of the thresholds $R_{0}$, $R_{p}$, $R_{0}^{eip}$, $R_{p}^{eip}$ and the number of malaria-infected humans $I_{h}$ are presented for different values of the EIP

The results demonstrated that the extrinsic incubation period decreases both the threshold for malaria persistence and the spread of malaria, (whether *Microsporidia MB* is present or not). Furthermore, the longer the extrinsic incubation period, the greater the reduction observed. This can be explained by the fact that a longer incubation period results in a higher number of exposed mosquitoes dying before they become infectious. Figure 7 illustrates how the thresholds and malaria incidence vary with different incubation periods.

Meanwhile, it is also important to note that the relationship that has been established between the estimated real malaria prevalence $p_{I}$ (which represents the equilibrium value of the proportion of malaria-infected humans) and the basic reproduction number $R_{0}$ as given in Eq. (31) remains unchanged, regardless of whether the extrinsic incubation period is included in the model. As a result, Fig. 5 holds true whether or not the extrinsic incubation period is considered.

## Results and discussion

### Results

The mathematical analysis consists of the determination of the basic reproduction number and a rigorous analysis of the stability of the model’s equilibria. In terms of the dynamics of *Microsporidia MB*, we have identified a stable state of complete *MB* infection. Regarding the *Microsporidia* dynamics, we obtain an *MB*-complete infection state stable when $\beta _{v} = 1$, an *MB*-free state stable when ($R_{1} < 1$) and a wild & *MB* coexistence state when ($R_{1} > 1$). Our findings highlight that perfect vertical transmission efficiency is essential to achieve a 100% infection rate among mosquitoes with *MB*-infected mosquitoes, leading to the desired population replacement. Furthermore, through numerical analysis, we discovered that achieving an $R_{1} > 1$, a marker for *MB* persistence in the mosquito population, requires a vertical transmission efficiency of no less than 55%, with horizontal transmission efficiency considered in the 0 to 0.5 range. Furthermore, numerical analysis to estimate the parameters that lead to a prevalence of *MB*-infected mosquitoes lower than 15% give some ranges of vertical and horizontal transmission efficiencies (Table 5 and Fig. 5), which are almost similar to values reported from data experiment (Herren et al 2020; Nattoh et al 2021). This finding justifies the low prevalence of *MB*-infected mosquitoes observed in nature (Herren et al 2020).

The malaria dynamics in absence of *Microsporidia* present a malaria-free state stable when $R_{0} < 1$ and a stable malaria-endemic state when $R_{0} > 1$. For the dynamics of malaria in the presence of *Microsporidia*, we get a second stable state without malaria when $R_{p} < 1$ and a stable state with malaria endemic when $R_{p} > 1$, with $R_{p} < R_{0}$. Essentially, the presence of the symbiont reduces the threshold for malaria elimination, which threshold is even decreasing with increasing *Microsporidia* prevalence in the mosquito population. In addition, the level of malaria incidence reduction associated with a given prevalence of *MB*-infected mosquitoes depends on the area’s initial level of transmission. To assess to what extent the presence of *MB* in the mosquito population can help to control malaria, we consider the actual malaria prevalence in various areas in Kenya and provide an estimation of the effect of the presence of *MB*-infected mosquitoes in Fig. 6. We observe from these results that malaria control can be achieved in the Highland with an average equilibrium prevalence of *MB*-infected mosquitoes of 15%, in the coast region with 40% and in the Lake region with 70%. Thus, the presence of *MB*-infected mosquitoes reduces malaria transmission and malaria control with *MB*-infected mosquitoes is easily achievable in areas with low malaria transmission. A similar conclusion was made concerning the spread of *Wolbachia* in the population of Aedes Aegypti population (Hughes and Britton 2013).

### Discussion

This model distinguishes itself by weaving together essential elements into an all-encompassing framework, shedding light on both *Microsporidia MB* dynamics within mosquito populations and their previously unexplored interactions with humans. This enhancement follows our previous work (Mfangnia et al 2023) offering an enriched narrative on malaria transmission pathways.

In this investigation, we crafted a sophisticated system of differential equations aimed at dissecting the transmission dynamics of *Microsporidia MB* among Anopheles mosquito populations and its consequent impact on malaria prevalence. Our primary goal was to probe the practicality of employing *Microsporidia MB* as a novel strategy for malaria management. To this end, we built a mathematical model to analyze the interactive dynamics of the humans, the mosquito population, the endosymbiont *MB* and the *Plasmodium*. This model highlights essential conditions under which malaria can either persist or be eliminated both in the absence and presence of *Microsporidia MB*. This model deliberately simplifies certain aspects, such as assuming a stable human population size, disregarding immunity and neglecting the *Plasmodium* incubation phase, yet it incorporates a carrying capacity to curb mosquito population growth. In addition, the model concentrates on the survival and dissemination patterns of *Microsporidia MB* in the absence of *Plasmodium*, incorporating critical observations on its transmission, mating behaviors, demographic structures, and the symbiont’s influence on mosquito vitality and reproduction, as documented by (Herren et al 2020; Nattoh et al 2021). Moreover, we included a lag time for *Plasmodium* neutralization by *Microsporidia MB*-infected mosquitoes, thus rendering them ineffective in furthering malaria transmission. This model paves the way for a nuanced understanding of the interplay between *Microsporidia MB* and malaria dynamics, offering a novel lens through which to view potential malaria control mechanisms.

The potential of integrated management strategies to curtail the prevalence of *MB*-infected mosquitoes and thereby facilitate malaria control warrants emphasis. Evidence underscores that the deployment of long-lasting insecticidal nets (LLINs) and Insect Residual Spraying (IRS) has effectively reduced the malaria basic reproduction number. Nevertheless, the integration of such strategies with the release of *MB*-infected mosquitoes demands cautious evaluation. The over-reliance on insecticidal interventions has been documented to escalate insecticide resistance, potentially nullifying the advantages accrued from their prior use (Mohammed-Awel et al 2020). This dilemma presents an avenue for subsequent research. Additionally, this study deliberately omits the exploration of vertical and horizontal transmission efficiencies of *MB* on malaria incidence, given the inherent challenge in biologically modifying these efficiencies and their correlation with low *MB*-infected mosquito prevalence in field studies (Herren et al 2020). Instead, we propose augmenting field prevalence through the lab-based amplification of *MB*-infected mosquito populations. The inherent biological and economic feasibility of *Microsporidia MB* for laboratory cultivation and its self-sustaining nature via transmission dynamics suggest that continuous releases might not be imperative. Future endeavor will aim to devise optimal, cost-effective, and time-efficient strategies for *MB*-infected mosquito releases, with a focus on attaining a sustainably manageable prevalence of *MB*-infected mosquitoes.

This study explores the effect of *MB*-infected mosquitoes on malaria incidence while neglecting the extrinsic incubation period. To assess the potential bias introduced by omitting this factor, we incorporate the extrinsic incubation period by introducing, in the equations describing the dynamics of malaria-infected humans, a delay before exposed mosquitoes can infect humans. The analysis revealed that, in general, neglecting the extrinsic incubation period results in an overestimation of both malaria cases and the associated threat. However, we also demonstrated that the specific predictions, given in this work, remain consistent regardless of whether the extrinsic incubation period is included.

Many assumptions are included in the model, such as, assuming that all malaria vectors are susceptible to *Microsporidia* infection. However, empirical research by (Nattoh et al 2021) and (Akorli et al 2021) identifies *Microsporidia MB*’s presence exclusively within *Anopheles Arabiensis*, *Funestus*, and *Gambiae* populations. Consequently, while our findings retain applicability in regions such as Kenya, where these species are predominant vectors (Okara et al 2010), the generalization of our results to areas dominated by other *Anopheles* species necessitates additional investigation. Moreover, this study assumes a uniform spatial distribution of both *MB*-infected and non-infected mosquitoes, an oversimplification that may not accurately reflect natural conditions. Furthermore, this model assumes no loss of *MB*-infection, perfect *Plasmodium* transmission-blocking, and disregards immunity. A more comprehensive mathematical analysis incorporating factors such as loss of *MB*-infection, imperfect *Plasmodium* transmission-blocking and malaria reinfections could provide deeper insights. The influence of environmental factors, such as seasonal variations, temperature, humidity, and precipitation on mosquito survival and birth rates (Chandra and Mukherjee 2022), presents another dimension for consideration in expanding our model. Such enhancements would afford more nuanced insights into *Microsporidia*’s dissemination and its consequent effects on malaria transmission. Additionally, given the nascent state of *Microsporidia MB* research, future mathematical explorations could benefit from incorporating spatial dynamics through partial differential equations and introducing variability via stochastic models, broadening the scope of our understanding of *Microsporidia MB* dynamics.

## Data Availability

All data generated or analysed during this study are included in this published article.

## Appendix A: Computation of equilibria

The equilibria are obtained by solving:

$$\begin{aligned} \dfrac{dF_{s}}{dt} = \dfrac{dF_{m}}{dt} = \dfrac{dF_{p}}{dt} = \dfrac{dF_{mp}}{dt} = \dfrac{dM_{s}}{dt} = \dfrac{dM_{m}}{dt} = \dfrac{dI_{h}}{dt} = 0.\end{aligned}$$

At equilibrium, the total male and female population sizes verify:

A1 $$\begin{aligned} \left\{ \begin{array}{l} \dfrac{dF_{T}}{dt} = bF_{T}\left( 1-\dfrac{F_{T}}{K}\right) - d_{f}F_{T} = 0; \\ \\ \dfrac{dM_{T}}{dt} = bF_{T}\left( 1-\dfrac{F_{T}}{K}\right) - d_{m}M_{T} = 0. \end{array} \right. \end{aligned}$$

Thus, considering $F_{T}, \ M_{T} \ne 0$, we have:

$$\begin{aligned}F_{T} = T_{f} = K\left( 1 - \dfrac{d_{f}}{b}\right) \ \text {and} \ T_{m} = \dfrac{d_{f}T_{f}}{d_{m}};\end{aligned}$$

Denoting by $F_{1} = F_{s} + F_{p}$ and $F_{2} = F_{m} + F_{mp}$, and expressing $M_{m}$ from $dM_{m}/dt = 0$, we have:

A2 $$\begin{aligned} \left\{ \begin{array}{l} new^{w} - new^{w}\beta _{h}^{mf}\epsilon _{m}\dfrac{M_{m}}{M_{T}} - d_{f}F_{1} = 0 \\ \\ new^{m} + new^{w}\beta _{h}^{mf}\epsilon _{m}\dfrac{M_{m}}{M_{T}} - d_{f}F_{2} = 0 \\ \\ M_{m} = \dfrac{new^{m}(1 + \beta _{h}^{fm}\epsilon _{w})T_{m}}{d_{m}T_{m} + new^{m}\beta _{h}^{fm}\epsilon _{w}}; \end{array} \right. \end{aligned}$$

where $$new^{m} = \beta _{v}d_{f}F_{2}$$ and $$new^{w} = d_{f}T_{f} - \beta _{v}d_{f}F_{2}$$.

Solving (A2), we have three possible solutions:

### Remark 3

If $\beta _{v} = 1$, we have ($F_{1} = 0$ and $F_{2} = T_{f}$). If $\beta _{v} < 1$, then we have either:

$$\begin{aligned} \begin{array}{rcl} \left( \begin{array}{c} F_{1} = T_{f} \\ \text {and} \\ F_{2} = 0 \end{array} \right) & or& \left( \begin{array}{c} F_{1} = T_{f}(R_{p}^{2}/R_{0}^{2}) = \tilde{F_{s}} \\ \text {and} \\ F_{2} = T_{f}(1 - R_{p}^{2}/R_{0}^{2}) = \tilde{F_{m}} \end{array} \right) . \end{array} \end{aligned}$$

1. If $\beta _{v} = 1$, we have $F_{1} = 0$ and $F_{2} = T_{f}$. Explicitly, $F_{s} = F_{p} = 0$ and $F_{m} + F_{mp} = T_{f}$. We easily deduce the following values:
  $$\begin{aligned} \left\{ \begin{array}{rcl} F_{s}^{01^{c}} &\, =\, & 0, \ F_{m}^{01^{c}} = T_{f}, \ F_{p}^{01^{c}} = 0, \ F_{mp}^{01^{c}} = 0, \\ \\ M_{s}^{01^{c}} &\, =\, & 0, \ M_{m}^{01^{c}} = T_{m}, \ I_{h}^{01^{c}} = 0. \end{array} \right. \end{aligned}$$
  This corresponds to the *disease-free with complete MB-infection equilibrium *$E^{01^{c}}$(*existing whenever *$\beta _{v} = 1$).
2. If $\beta _{v} < 1$, $F_{1} = T_{f} \ \text {and} \ F_{2} = 0$. Explicitly, $F_{s} + F_{p} = T_{f}$ and $F_{m} = F_{mp} = 0$. We distinguish two subcases: either $F_{p} = 0$ and $F_{s} = T_{f}$ or $0< F_{p} < T_{f}$.
  - $F_{p} = 0$ and $F_{s} = T_{f}$ In this case, it is easy to observe that:
    $$\begin{aligned}F_{p} = 0 \Rightarrow I_{h} = 0; \ \text {and} \\F_{m} = F_{mp} = 0 \Rightarrow M_{m} = 0 \ \text {and} \ M_{s} = T_{m}.\end{aligned}$$
    Thus, we obtain the following equilibrium:
    $$\begin{aligned} \left\{ \begin{array}{rcl} F_{s}^{00} &\, =\, & T_{f}, \ F_{m}^{00} = 0, \ F_{p}^{00} = 0, \ F_{mp}^{00} = 0; \\ \\ M_{s}^{00} &\, =\, & T_{m}, \ M_{m}^{00} = 0, \ I_{h}^{00} = 0. \end{array} \right. \end{aligned}$$
    This is the *disease-free with wild mosquitoes equilibrium* $E^{00}$ (*that always exists*).
  - $0< F_{p} < T_{f}$ Using $dI_{h}/dt = 0$ and $dF_{p}/dt = 0$, we get:
    $$\begin{aligned} \left\{ \begin{array}{l} I_{h} = \dfrac{\eta p^{mh}F_{p}N_{0}}{\eta p^{mh}F_{p} + \gamma _{h}N_{0}}; \\ \\ \dfrac{\eta p^{hm}I_{h}F_{s}}{N_{0}} - d_{f}F_{p} = 0. \end{array} \right. \end{aligned}$$
    Replacing the first expression in the second, we have:
    A3 $$\begin{aligned} F_{p} = \dfrac{\eta ^{2}p^{hm}p^{mh}F_{s} - d_{f}\gamma _{h}N_{0}}{d_{f}\eta p^{mh}}. \end{aligned}$$
    Then, considering $F_{s} + F_{p} = T_{f}$, we get:
    $$\begin{aligned} {\left\{ \begin{array}{ll} F_{s} &\, =\, \dfrac{T_{f}(\eta p^{hm}+d_{f}R_{0}^{2})}{R_{0}^{2}(\eta p^{hm}+d_{f})}; \\ \\ F_{p} &\, =\, \dfrac{\eta p^{hm}T_{f}(R_{0}^{2}-1)}{R_{0}^{2}(\eta p^{hm}+d_{f})}. \end{array}\right. } \end{aligned}$$
    By observing that $F_{m} = F_{mp} = 0$, we easily deduce that $M_{m} = 0, \ M_{s} = T_{m}$, and the equilibrium is defined as follows:
    A4 $$\begin{aligned} {\left\{ \begin{array}{ll} F_{s}^{10} &\, =\, \dfrac{T_{f}(\beta _{1}+d_{f}R_{0}^{2})}{R_{0}^{2}(\beta _{1}+d_{f})}, \ F_{m}^{10} = 0, \\ \\ F_{p}^{10} &\, =\, \dfrac{\beta _{1}T_{f}(R_{0}^{2}-1)}{R_{0}^{2}(\beta _{1}+d_{f})}, \ F_{mp}^{10} = 0, \\ \\ M_{s}^{10} &\, =\, T_{m}, \ \ M_{m}^{10} = 0, \\ \\ I_{h}^{10} &\, =\, \dfrac{d_{f}N_{0}(R_{0}^{2}-1)}{d_{f}R_{0}^{2}+\beta _{1}}. \end{array}\right. } \end{aligned}$$
    This is the *endemic and MB-free equilibrium* $E^{10}$ (*existing whenever *$R_{0} > 1$).
3. We consider $$\beta _{v} < 1$$,
  $$\begin{aligned} {\left\{ \begin{array}{ll} F_{1} &\, =\, \dfrac{T_{f}(R_{1} - 1)}{\beta _{v}(R_{1} + \beta _{h}^{fm}\epsilon _{w})} = T_{f}\dfrac{R_{p}^{2}}{R_{0}^{2}} = \tilde{F_{s}}; \\ \\ F_{2} &\, =\, T_{f}\left( 1 -\dfrac{R_{p}^{2}}{R_{0}^{2}}\right) = \tilde{F_{m}}. \end{array}\right. } \end{aligned}$$
  Then, we can compute the values for $M_{s}$ and $M_{m}$. Recalling the expression of $M_{m}$, in (A2), we have:
  $$\begin{aligned}M_{m} = \dfrac{new^{m}(1 + \beta _{h}^{fm}\epsilon _{w})T_{m}}{d_{m}T_{m} + new^{m}\beta _{h}^{fm}\epsilon _{w}};\end{aligned}$$
  where $new^{m} = \beta _{v}d_{f}\tilde{F_{m}}.$ This expression can be easily reduced and we get:
  $$\begin{aligned}M_{m} = T_{m}\left( 1 - \dfrac{1}{R_{1}}\right) \ \text {and} \ M_{s} = \dfrac{T_{m}}{R_{1}}.\end{aligned}$$
  For the computation of the equilibria values in the female mosquito populations, we distinguish two subcases: either $F_{p} = 0$ or $F_{p} \ne 0$.
  1. Case $$F_{p} = 0$$.
    We have $F_{p} = 0 \Rightarrow I_{h} = 0 \ \text {and} \ F_{mp} = 0$. So, $F_{s} = \tilde{F_{s}}$ and $F_{m} = \tilde{F_{m}}$. Therefore, we get the following equilibrium:
    A5 $$\begin{aligned} {\left\{ \begin{array}{ll} F_{s}^{01} &\, =\, \dfrac{T_{f}R_{p}^{2}}{R_{0}^{2}}, \ F_{m}^{01} = \dfrac{T_{f}(R_{0}^{2}-R_{p}^{2})}{R_{0}^{2}}, \\ \\ F_{p}^{01} &\, =\, 0, \ F_{mp}^{01} = 0, \ M_{s}^{01} = \dfrac{T_{m}}{R_{1}},\\ \\ M_{m}^{01} &\, =\, T_{m}\left( 1-\dfrac{1}{R_{1}}\right) , \ I_{h} = 0. \end{array}\right. } \end{aligned}$$
    This is the *disease-free with wild and MB-infected mosquitoes* equilibrium $E^{01}$ (*existing whenever *$R_{1} > 1\textit{ and }R_{2} < 1$).
  2. Case $$F_{p} \ne 0$$.
    Here, it is enough to express $F_{s}$ as a function of $F_{p}$ and $F_{m}$ as a function of $F_{mp}$ to get the values of $F_{s},\ F_{m}, \ F_{p} \ \text {and} \ F_{mp}$. -The relation between $F_{s}$ and $F_{p}$ has already been established in (A3). Let $\beta _{1}$ be $\eta p^{hm}$. By considering $F_{s} + F_{p} = \tilde{F_{s}}$, and observing that $\gamma _{h}N_{0}R_{0}^{2}/\eta p^{mh} = \eta p^{hm}T_{f}/d_{f}$, we easily get:
    $$\begin{aligned} {\left\{ \begin{array}{ll} F_{s} &\, =\, \dfrac{T_{f}(\beta _{1}+d_{f}R_{p}^{2})}{R_{0}^{2}(\beta _{1}+d_{f})}; \\ \\ F_{p} &\, =\, \dfrac{\beta _{1}T_{f}(-1+R_{p}^{2})}{R_{0}^{2}(\beta _{1}+d_{f})}. \end{array}\right. } \end{aligned}$$
    On the other hand, we have from the expression $dF_{mp}/dt = 0$,
    $$\begin{aligned}F_{mp} = \dfrac{\eta p^{hm}I_{h}F_{m}}{(\alpha + d_{f})N_{0}},\end{aligned}$$
    with
    $$\begin{aligned}I_{h} = \dfrac{\eta p^{mh}F_{p}N_{0}}{\eta p^{mh}F_{p} + \gamma _{h}N_{0}} = \dfrac{d_{f}(-1 + R_{p}^{2})N_{0}}{d_{f}R_{p}^{2} + \beta _{1}}.\end{aligned}$$
    By considering $F_{m} + F_{mp} = \tilde{F_{m}}$, we get the expressions of $F_{m}$ and $F_{mp}$ as summarized in the following:
    A6 $$\begin{aligned} {\left\{ \begin{array}{ll} F_{s}^{11} &\, =\,\dfrac{T_{f}(\beta _{1}+d_{f}R_{p}^{2})}{R_{0}^{2}(\beta _{1}+d_{f})}; \ F_{p}^{11} =\dfrac{\beta _{1}T_{f}(-1+R_{p}^{2})}{R_{0}^{2}(\beta _{1}+d_{f})};\\ \\ F_{m}^{11} &\, =\,\dfrac{(d_{f}+\alpha )(\beta _{1}+d_{f}R_{p}^{2})\tilde{F_{m}}}{(d_{f}+\alpha )(\beta _{1}+d_{f}R_{p}^{2})+\beta _{1}d_{f}(-1+R_{p}^{2})};\\ \\ F_{mp}^{11} &\, =\,\dfrac{\beta _{1}d_{f}(-1+R_{p}^{2})\tilde{F_{m}}}{(d_{f}+\alpha )(\beta _{1}+d_{f}R_{p}^{2})+\beta _{1}d_{f}(-1+R_{p}^{2})}; \\ \\ M_{s}^{11} &\, =\, \dfrac{T_{m}}{R_{1}}; \ M_{m}^{11} = T_{m}\left( 1-\dfrac{1}{R_{1}}\right) ; \\ \\ I_{h}^{11} &\, =\, \dfrac{d_{f}N_{0}(-1+R_{p}^{2})}{\beta _{1}+d_{f}R_{p}^{2}}. \end{array}\right. } \end{aligned}$$
    This is the *complete coexisting equilibrium *$E^{11}$ (existing whenever $R_{1} > 1$, $R_{2} < 1$ and $R_{p} > 1$).
